# Smu1 and RED are required for activation of spliceosomal B complexes assembled on short introns

**DOI:** 10.1038/s41467-019-11293-8

**Published:** 2019-08-13

**Authors:** Sandra Keiper, Panagiotis Papasaikas, Cindy L. Will, Juan Valcárcel, Cyrille Girard, Reinhard Lührmann

**Affiliations:** 1 Department of Cellular Biochemistry, Max Planck Institute for Biophysical Chemistry, Am Fassberg 11, 37077 Göttingen, Germany; 2 Centre de Regulació Genòmica, The Barcelona Institute of Science and Technology and Universitat Pompeu Fabra, Dr. Aiguader 88, 08003 Barcelona, Spain; 3 Institució Catalana de Recerca i Estudis Avançats (ICREA), Pg. Lluís Companys, 08010 Barcelona, Spain; 4 Friedrich Miescher Institute for Biomedical Research (FMI), Maulbeerstrasse 66, 4058 Basel, Switzerland; 5 Swiss Institute of Bioinformatics, 4058 Basel, Switzerland

**Keywords:** Genome informatics, Genetics, RNA splicing, RNA

## Abstract

Human pre-catalytic spliceosomes contain several proteins that associate transiently just prior to spliceosome activation and are absent in yeast, suggesting that this critical step is more complex in higher eukaryotes. We demonstrate via RNAi coupled with RNA-Seq that two of these human-specific proteins, Smu1 and RED, function both as alternative splicing regulators and as general splicing factors and are required predominantly for efficient splicing of short introns. In vitro splicing assays reveal that Smu1 and RED promote spliceosome activation, and are essential for this step when the distance between the pre-mRNA’s 5′ splice site (SS) and branch site (BS) is sufficiently short. This Smu1-RED requirement can be bypassed when the 5′ and 3′ regions of short introns are physically separated. Our observations suggest that Smu1 and RED relieve physical constraints arising from a short 5′SS-BS distance, thereby enabling spliceosomes to overcome structural challenges associated with the splicing of short introns.

## Abstract (web summary)

Human spliceosome components Smu1 and RED regulate alternative splicing. Here the authors show that Smu1 and RED are also required for constitutive splicing of short introns.

## Introduction

Spliceosome assembly is initiated by the stepwise recruitment of the U1 and U2 snRNPs to the 5′ splice site (SS) and branch site (BS), respectively, of an intron, generating the spliceosomal A complex. Subsequently, the preformed U4/U6.U5 tri-snRNP binds^1,2^, yielding the B complex. Profound compositional and conformational rearrangements, including loss of U4 and the exchange of more than 35 proteins, lead to the activated B^act^ complex. Spliceosome activation is a multi-step process that is initiated by unwinding of the U4/U6 duplex by the RNA helicase Brr2. This allows U6 to rearrange and form the functionally important U6 internal stem loop (ISL), and to establish new base pairing interactions with U2 to form key catalytically active U2/U6 RNA structures in the spliceosome. However, the roles of other spliceosomal proteins in the complex activation process, especially in higher eukaryotes, remains unclear. Subsequent remodelling by the Prp2 helicase generates the B* complex, which catalyses step 1 of pre-mRNA splicing, i.e. 5′SS cleavage and intron lariat-3′ exon formation. The C complex, formed during step 1, is activated to the C* complex, which catalyses intron excision and ligation of the 5′ and 3′ exons (step 2)^2^.

Most of the ~280,000 annotated human introns are longer than 70 nucleotides (nts), with only a small fraction (0.25%) shorter than 73 nts^3^. Previous studies showed that intron length affects the efficiency of splicing, with those introns shorter than 80 nts displaying a clear reduction in splicing^4,5^. Moreover, a minimal distance separating the 5′SS and BS—estimated to be ca 50 nts—is required to allow efficient spliceosome assembly and splicing^4–9^. This minimal distance appears to be needed for the simultaneous and productive binding of snRNPs to the 5′ and 3′ regions of the intron, which otherwise would be sterically hindered. Indeed, a 5′SS–BS distance of 50 nts allows the assembly of early spliceosomal complexes on the adenovirus E1A pre-mRNA, but if shortened to 35 nts, assembly is completely abolished^7^. The distance between the 5′SS and BS can also affect alternative splicing events, as shown for the mutually exclusive splicing of exons of the alpha-tropomyosin pre-mRNA^9^.

During B complex formation, several B-specific proteins (i.e. RED, MFAP1, FBP21, Smu1, Prp38, Snu23, UBL5, NPW38 and NPW38BP) interact with the spliceosome and are released/destabilised already during the subsequent activation stage^10^. Several B-specific proteins, including Smu1 and RED, have no orthologs in the yeast *Saccharomyces cerevisiae*, suggesting that their roles in splicing are confined to regulatory tasks rather than essential core functions. At least one of the B-specific proteins, namely Prp38, is required for spliceosome activation in yeast^11^ and was suggested to play a similar role in humans^12^. The B-specific proteins, Smu1 and RED, are conserved in higher eukaryotes and they have been shown to modulate alternative splicing in various organisms^13–18^. Smu1 and RED interact with each other^13,16,19–21^ and they appear to directly stabilise each other within the cell and function as a unit^16,19^. Smu1 and RED have been implicated in other cellular processes aside from pre-mRNA splicing, including mitotic spindle integrity^22,23^, genome stability^18,24–26^, regulating mitotic kinases/phosphatases^27^, aiding MAD1 localisation to kinetochores^28^ and binding to influenza virus RNA polymerase^19^. Based on structural and yeast two-hybrid studies, Smu1 and RED were proposed to act as a binding platform that mediates multiple protein–protein interactions within the spliceosome^20,21^. The recent cryogenic electron microscopy (cryo-EM) structure of the human B complex shed light on possible roles for Smu1 and RED, indicating that they play an important structural role in bridging Brr2 with the U2 protein SF3B3^29^. This observation challenges the notion that RED and Smu1 solely play regulatory roles, suggesting instead a core structural function during spliceosome assembly.

Knockdown of Smu1 or RED in human cells significantly changes alternative splicing patterns^15^. However, it was unclear whether Smu1 and RED also play a role in constitutive splicing. Here, we investigate the roles of the human Smu1 and RED proteins in splicing and spliceosome assembly. We show that RED and Smu1 knockdown not only affects alternative splicing outcomes in vivo, but also leads to the retention of constitutively-spliced introns, the majority of which are relatively short. We also demonstrate that Smu1 and RED are important for efficient splicing in vitro, and that the splicing of short introns with a minimum 5′SS–BS distance is abolished when Smu1 and RED are absent. Our data demonstrate that Smu1 and RED play important roles during spliceosome activation, and likely help to relieve structural constraints that hinder activation of spliceosomes formed on introns with a short 5′SS–BS distance.

## Results

### Smu1 and RED knockdown hinders the splicing of short introns

**Fig. 1:**
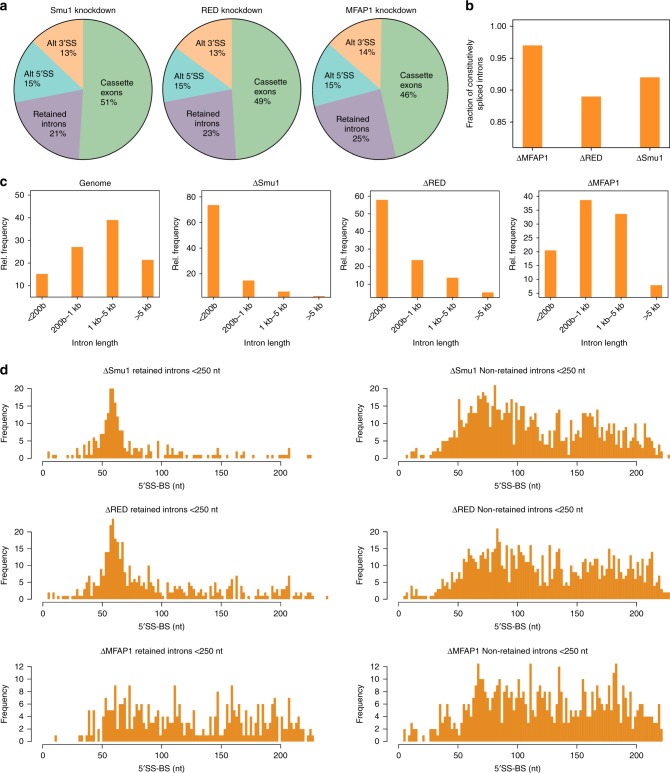
Knockdown of Smu1 and RED leads to the retention of constitutively spliced introns. **a** Pie charts showing the distribution of different splicing events altered in HeLa cells after knockdown of Smu1, RED or MFAP1, as determined by RNA-seq. Usage of alternatively spliced (Alt) 3′ or 5′ splice sites, changes in the inclusion or skipping of cassette exons, and retention of introns are indicated. **b** Fraction of constitutively spliced introns among those retained (unspliced) upon knockdown of Smu1, RED or MFAP1. **c** Introns were sorted into 4 classes according to their length—i.e. <200 bases, 200–1 Kb, 1–5 Kb and >5 Kb. Charts showing the relative distribution of these 4 intron length classes in the genome or in those introns retained after Smu1, RED or MFAP1 knockdown. **d** 5′SS to BS distances in short introns (<250 nts long) retained after Smu1, RED or MFAP1 knockdown versus non-retained short introns with a matching intron-length distribution. The observed distributions for retained versus non-retained (unaffected) short introns are significantly different for ΔSmu1 and ΔRED (*p*-value < 1e−12, two-sided, two sample Smirnov–Kolmogorov test), but not for ΔMFAP1 (*p*-value > 0.01)

To elucidate the role of Smu1 and RED, we investigated global effects on pre-mRNA splicing after their knockdown in HeLa cells using RNAi followed by RNA-Seq analysis. Cells were transfected with siRNAs specific for Smu1, RED or, for comparison, the B complex-specific protein MFAP1. Total RNA was harvested 50 h post transfection and subjected to paired-ended Illumina sequencing. Western blotting revealed a 70–80% decrease in Smu1, RED or MFAP1 in the knockdown cells compared to control cells (Supplementary Fig. 1a), and that knockdown of Smu1 leads to a substantial decrease in RED and vice versa (Supplementary Fig. 1b). Consistent with previous results obtained using multiplexed reverse transcription polymerase chain reaction (RT-PCR) analysed by capillary electrophoresis (LabChip)^15^, Smu1 or RED depletion led to changes in the alternative splicing (AS) of cassette exons (Cex) (Fig. 1a), the majority of which were skipped (Supplementary Fig. 1c) or in the selection of alternative 5′ and 3′ splice sites (Fig. 1a). Strikingly, 21–23% of the changes in splicing observed in ΔSmu1 and ΔRED cells involved the retention of introns (RI), the vast majority (>90%) of which are constitutively-spliced introns (Fig. 1b, Supplementary Data 1). This demonstrates that Smu1 and RED also function as more general splicing factors. MFAP1 knockdown also affected alternative and constitutive splicing to a similar extent, but in many cases led to changes in the splicing of different subsets of introns (Supplementary Fig. 1d), suggesting differential roles for the B-specific proteins during splicing (Fig. 1a, c). Consistent with Smu1 and RED functioning as a unit during constitutive splicing, the overlap of introns retained after either Smu1 or RED knockdown was strikingly high (75%), whereas the overlap between knockdown of either of them and that of MFAP1 was only ca 25% (Supplementary Fig. 1d).

Close inspection of the introns retained upon Smu1 or RED knockdown did not reveal common sequence motifs, or features such as a higher or lower percent of GC nucleotides. However, the majority of these introns were relatively short. In ΔSmu1 and ΔRED cells, approximately 60% of the retained introns were shorter than 200 nts, although such short introns represent only ~15% of introns annotated in the human genome (Fig. 1c). In comparison, only 20% of introns retained upon MFAP1 knockdown were shorter than 200 nts (Fig. 1c). Furthermore, introns with the highest retention levels in ΔSmu1 and ΔRED cells had, in most cases, lengths between 75 and 100 nts (Supplementary Fig. 1e). Close inspection of the distribution of distances between the 5′SS and BS showed that short introns retained upon Smu1 or RED knockdown display a peak between 50 and 70 nts, while short introns unaffected by Smu1/RED depletion displayed a wider distribution of 5′SS–BS distances, as was also the case for short introns either retained or unaffected by MFAP1 knockdown (Fig. 1d, Supplementary Fig. 1f). Thus, we conclude that a short 5′SS–BS distance is a feature associated with the sensitivity of introns to depletion of Smu1 and RED in vivo.

### Shortening the 5′SS–BS distance inhibits splicing in vitro

**Fig. 2:**
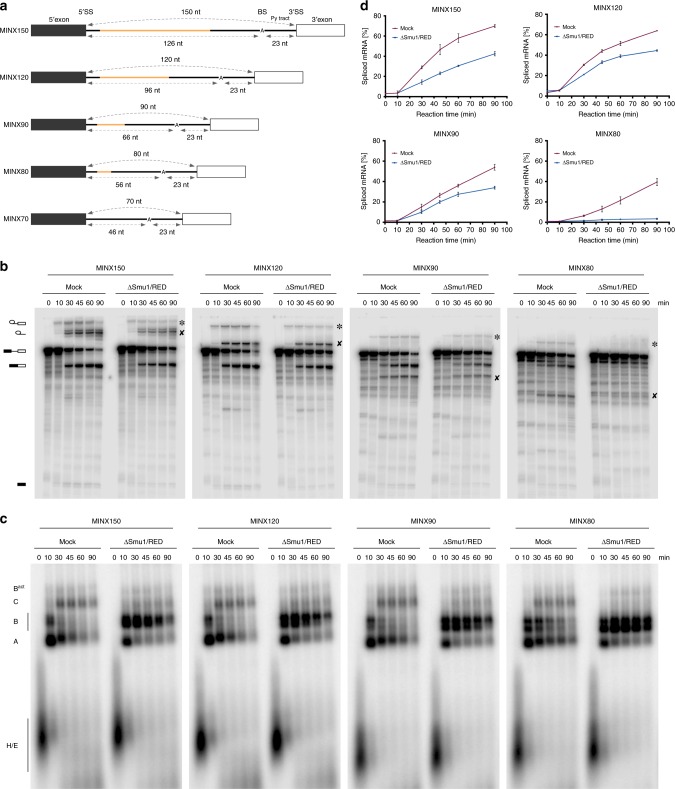
In the absence of Smu1/RED splicing is abolished when the 5′SS–BS distance is short. **a** Schematic of MINX-MS2 pre-mRNAs with varying intron lengths. The MINX120 intron (120 nt) was extended by 30 nt (MINX150) or truncated by 30 nt (MINX90), 40 nt (MINX80) or 50 nt (MINX70). Variable regions are illustrated in orange. Reactive sites (5′SS, BS, 3′SS) and the PY tract remained unchanged. **b**, **c** Kinetics of in vitro splicing (**b**) and spliceosome assembly (**c**) in the presence or absence of Smu1/RED. ^32^P-labelled MINX pre-mRNAs were incubated under splicing conditions in the presence of mock-depleted (i.e. treated in an identical manner but without antibody) or Smu1/RED-immunodepleted HeLa nuclear extract for 0–90 min. In panel (**b**), RNA was analysed by denaturing PAGE and visualised by autoradiography. The pre-mRNA and splicing intermediates and products are indicated on the left. The asterisk and ‘x’ indicate the intron-3′exon intermediate or spliced-out intron of the various pre-mRNAs, which migrate differently due to their varying sizes. In panel (**c**), spliceosomal complex formation was analysed on a native agarose gel and visualised by autoradiography. The positions of the H/E, A, B, C and B^act^ complexes are indicated on the left. **d** Quantification of the percent of spliced mRNA formed in mock-depleted (red line) or Smu1/RED-depleted extract (blue line) at different time points with the indicated MINX pre-mRNAs. Error bars represent the standard deviation obtained from two independent experiments. Source data are provided as a Source Data file

To dissect the role of Smu1 and RED in the splicing of short introns, we performed splicing in HeLa nuclear extract (NE) with MINX-MS2 pre-mRNA and either truncated or extended versions thereof (Fig. 2a). MINX pre-mRNAs with a 150, 90, 80 or 70 nts long intron (referred to as MINX150, -90, -80 and -70, respectively) were generated by removing or adding nts between the 5′SS and the branch site of MINX120, but without altering the 15 nts directly downstream of the 5′SS or the 20 nts upstream of the BS (Fig. 2a). As a result, the 5′SS is separated from the BS by 126, 96, 66, 56 and 46 nts in the MINX150, -120, -90, -80 and -70 pre-mRNAs, respectively. Increasing intron length had no appreciable effect on splicing (Supplementary Fig. 2a, c, compare MINX120 and MINX150). In contrast, shortening the MINX120 intron to 90 or 80 nts, and thus the 5′SS–BS distance, slowed down the kinetics of the splicing reaction. For example, 37% of the MINX90, and only 20% of MINX80 was spliced after 30 min, compared to ca 60% of MINX120 or MINX150 (Supplementary Fig. 2a, c). Strikingly, when the MINX intron was shortened to 70 nt, leading to a 5′SS–BS distance of 46 nts, splicing was abolished (Supplementary Fig. 2a, c). Native gel electrophoresis revealed a delay in the formation of B^act^ and catalytically-active C complexes on MINX90 and MINX80, which were detectable only after 30 min compared to detection at 10 min on MINX120 and MINX150 (Supplementary Fig. 2b). The A to B and B to B^act^ transitions were also less efficient with MINX80, and appeared to be severely impaired with MINX70 (Supplementary Fig. 2b), indicating that the assembly of the spliceosome was compromised. Our data are in agreement with previous studies indicating that a minimum distance between the 5′SS and BS of approximately 50 nts is required for efficient spliceosome assembly in vitro.

### Smu1/RED depletion hinders efficient spliceosome activation

To assess their roles in pre-mRNA splicing, we immunodepleted Smu1 and RED from HeLa nuclear extract using an anti-Smu1 antibody. Consistent with them forming a highly stable dimer, both Smu1 and RED were efficiently immunodepleted, whereas the levels of other B-specific proteins such as MFAP1, FBP21 or Prp38, and of core spliceosomal factors (Snu114, Snu66 and SF3A2) were largely unaffected (Supplementary Fig. 3). Splicing of MINX150, MINX120, MINX90 and MINX80 pre-mRNA was substantially reduced in the ΔSmu1/RED versus mock-depleted extract (Fig. 2b, d). For example, the amount of mRNA generated with the MINX150, MINX120 and MINX90 pre-mRNAs was 71%, 62% and 56%, respectively, after 90 min in the mock-depleted NE, but dropped to 44%, 45% and 33%, respectively, in ΔSmu1/RED NE. Thus, Smu1 and RED are important for efficient splicing in vitro. The most dramatic effect was observed with MINX80, where splicing was essentially abolished in ΔSmu1/RED NE (Fig. 2b, d). Thus, when the 5′SS–BS distance reaches a critical lower limit, splicing becomes highly dependent on Smu1/RED. Analysis of spliceosome assembly revealed that depletion of Smu1 and RED led to an accumulation of B complexes compared to the mock, with all of the MINX pre-mRNAs tested (Fig. 2c). This indicates that the B to B^act^ transition is hindered in the absence of Smu1 and RED. This effect was most prominent with MINX80, where the strong accumulation of B complexes in ΔSmu1/RED extract remained constant over 90 min, suggesting that the activation of spliceosomes formed on introns with a short 5′SS–BS distance is blocked when Smu1 and RED are absent. Importantly, expanding the distance between the BS and 3′SS of MINX-80 by 10 nts did not relieve this block (Supplementary Fig. 4).

**Fig. 3:**
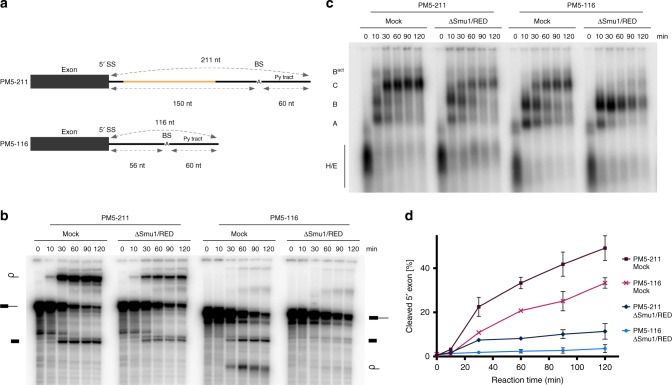
The distance between the 5′SS and BS determines whether splicing is Smu1/RED-dependent. **a** Schematic of PM5-211 and PM5-116 pre-mRNAs. The variable region is illustrated in orange. The lack of a 3′ exon stalls splicing after the first catalytic step (i.e. at the C complex). **b**, **c** Kinetics of in vitro splicing (**b**) and spliceosome assembly (**c**) in the presence or absence of Smu1/RED. ^32^P-labelled PM5-211 and PM5-116 were incubated under splicing conditions in the presence of mock-depleted or Smu1/RED-immunodepleted HeLa nuclear extract for 0–120 min. The pre-mRNA and splicing intermediates, as well as spliceosomal complex formation were analysed as in Fig. 2. **d** Quantification of the percent of spliced mRNA generated with the PM5-211 and PM5-116 pre-mRNAs in Mock- or ΔSmu1/RED-depleted extract. Error bars represent the standard deviation obtained from two independent experiments. Source data are provided as a Source Data file

Similar results were obtained with the PM5 pre-mRNA (PM5-211), which contains a 5′ exon followed by a 211 nts long intron, but lacks a 3′ exon (Fig. 3a), blocking splicing after step 1. When the distance between the 5′SS and BS of PM5 was shortened from 151 nts to 56 nts (i.e. the same distance as in MINX80), generating PM5-116, splicing was nearly abolished in the ΔSmu1/RED extract (Fig. 3b, d). There was also a clear accumulation of B complexes, although they appeared to be somewhat less stable at longer incubation times than those assembled on MINX80, and a concomitant reduction in B^act^/C complexes (Fig. 3c). These results show that despite its 116 nts long intron, spliceosome assembly on the PM5-116 pre-mRNA was also stalled at the B complex stage, indicating that the distance separating the 5′SS and the BS, and not the total intron length, determines whether Smu1 and RED are essential for spliceosome activation.

**Fig. 4:**
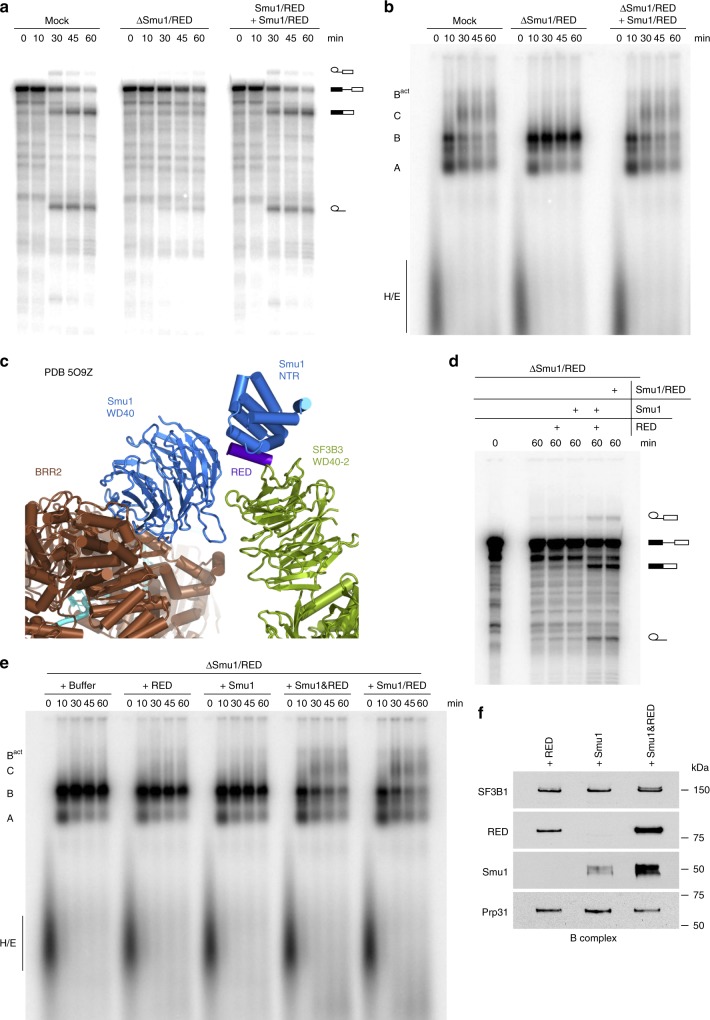
Restoration of splicing activity to ΔSmu1/RED extract requires the addition of both proteins. **a**, **b** In vitro splicing (**a**) and the assembly of spliceosomal complexes on MINX80 pre-mRNA (**b**) are restored after addition of purified Smu1/RED dimer to ΔSmu1/RED HeLa nuclear extract. The pre-mRNA and splicing intermediates and products, as well as spliceosomal complex formation were analysed as in Fig. 2. **c** Smu1 (blue) and RED (purple) bridge the U5 Brr2 helicase (brown) and U2 snRNP SF3B3 protein (green) in the human B complex. In the 3D model of the B complex generated by Cryo-EM^29^, only the N-terminal region (NTR) and C-terminal WD40 domain of Smu1, plus a short alpha helix of the RED protein that interacts with the Smu1 NTR, could be localised between Brr2 and the WD40-2 domain of SF3B3. **d**, **e** In vitro splicing (**d**) and spliceosome assembly (**e**) on MINX80 pre-mRNA after addition of either purified Smu1 or RED alone, both individual proteins (Smu1 and RED), or purified Smu1/RED dimer (as indicated above each gel). **f** Efficient incorporation of Smu1 or RED into spliceosomal B complexes requires the presence of both proteins. B complexes formed in ΔSmu1/RED HeLa nuclear after addition of purified Smu1 or RED alone or both proteins (as indicated above) were affinity purified and the presence of Smu1 and RED, or of SF3B1 and Prp31, which served as loading controls, was analysed by Western blot using antibodies against the proteins indicated on the left. Source data are provided as a Source Data file. Panel (**a**) was reprinted from Bertram, K. et al. Cryo-EM structure of a pre-catalytic human spliceosome primed for activation. *Cell* **170**, 701–713 (2017), with permission from Elsevier

Adding back purified Smu1/RED dimer (Supplementary Fig. 5b), to ΔSmu1/RED extract fully restored both splicing of the MINX80 pre-mRNA and spliceosome assembly (Fig. 4a, b). This confirms that the inhibitory effect on activation is due solely to the absence of Smu1/RED and not other co-depleted factors. Taken together, these data indicate that Smu1 and RED facilitate B^act^ formation, and are essential for the activation of spliceosomes assembled on introns with a short 5′SS–BS distance.

### Both proteins are required for splicing in ΔSmu1/RED extract

RED is a highly intrinsically-disordered protein, whereas Smu1 contains a C-terminal WD40 domain and an N-terminal region that interacts with RED^21^ (Supplementary Fig. 4a). Cryo-EM, which allowed the localisation of the N-terminal and WD40 domains of Smu1 but only a short α-helix of RED, revealed that Smu1 and RED interact in the human B complex, forming a bridge between the Brr2 helicase and U2 SF3B3 protein that involves Smu1’s WD40 domain (Fig. 4c). To determine whether Smu1 or RED alone suffice for efficient activation, we added each protein individually to the ΔSmu1/RED extract and, after a 15 min preincubation, analysed splicing of MINX80. When Smu1 or RED were added alone, the accumulation of B complexes persisted and no increase in splicing was observed (Fig. 4d, e). In contrast, the block in the B to B^act^ transition was alleviated and there was an increase in spliced mRNA when the purified Smu1/RED dimer, or both of the individual proteins were added together (Fig. 4d, e). This is consistent with the idea that both proteins act as a unit during splicing and that dimer formation is a prerequisite for the productive integration of each protein into the spliceosome. To test the latter, we purified B complexes formed on MINX80 pre-mRNA after addition of both Smu1 and RED, or of each protein alone, to ΔSmu1/RED extract. Only relatively low amounts of Smu1 or RED copurified with spliceosomes when added individually, whereas association of these proteins was greatly enhanced when both proteins were added together to the splicing reaction (Fig. 4f). Thus, Smu1 and RED are dependent on one another for productive association with spliceosomal B complexes. Dimerisation might introduce structural changes in Smu1 and/or RED that enable their proper/stable interaction with other B complex components. Indeed, Smu1 from *Caenorhabditis* *elegans* undergoes global conformational changes upon binding to RED^21^.

Deletion of the C-terminal WD40 domain of Smu1 did not affect Smu1/RED dimer formation, as evidenced by the copurification of the Smu1-ΔWD40 mutant together with His-tagged RED after affinity purification with Ni-NTA beads (Supplementary Fig. 5b). However, the Smu1-ΔWD40/RED dimer no longer copurified with spliceosomes (Supplementary Fig. 5e) and accordingly, failed to restore spliceosome assembly and the catalytic steps of splicing to the levels observed with the wildtype heterodimer (Supplementary Fig. 5c, d). Thus, Smu1’s WD40 domain plays an important role in the recruitment of Smu1 and RED to the B complex.

### Characterisation of ΔSmu1/RED spliceosomal B complexes

**Fig. 5:**
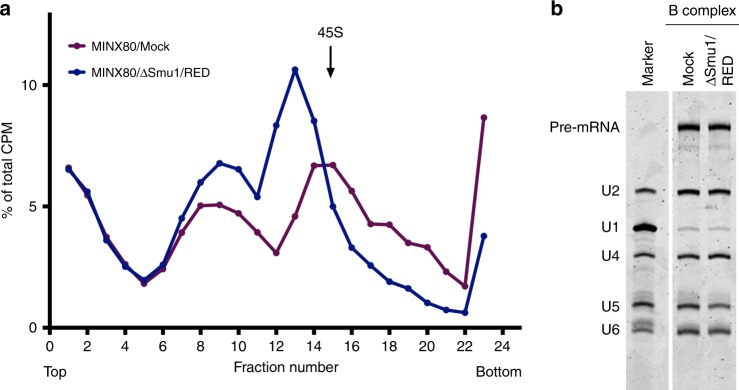
Characterisation of B complexes that accumulate in the absence of Smu1 and RED. **a** Glycerol gradient sedimentation profile of spliceosomal complexes assembled after 8 min on MINX80 in mock-depleted or Smu1/RED-depleted extract. The amount of the ^32^P-pre-mRNA in each gradient fraction was determined by Cherenkov counting. **b** RNA composition of B complexes assembled on MINX80 in mock- or Smu1/RED-depleted extract. Wildtype or ΔSmu1/RED spliceosomal complexes peaking in gradient fractions 14–16 or 12–14, respectively, were subjected to MS2 affinity purification, and the extracted RNA was analysed by denaturing PAGE followed by SYBR gold staining. The position of the pre-mRNA and UsnRNAs is shown on the left. Source data are provided as a Source Data file

To investigate whether Smu1 and RED depletion affects the recruitment of other spliceosomal proteins, we affinity-purified ΔSmu1/RED B complexes assembled on MINX80. For comparison, B complexes assembled in mock-depleted extract after 8 min (kinetically-stalled B complexes) were also purified. Gradient centrifugation of the 8 min splicing reactions prior to affinity selection showed that B complexes formed in mock-depleted extract peaked in fractions 14–16 (i.e. the 45S region of the gradient), while ΔSmu1/RED complexes peaked in fractions 12–14, and thus possess a smaller *S*-value (Fig. 5a). This could be due to the presence of fewer proteins/snRNAs and/or to a less compact structure. However, the snRNA composition of the ΔSmu1/RED complexes was identical to that of the kinetically-stalled B complexes, which contained stoichiometric amounts of the U2, U4, U5 and U6 snRNAs, and trace amounts of the U1 snRNA (Fig. 5b), which is characteristic for B complexes. The presence of U4 shows that the Brr2-mediated unwinding of the U4/U6 helix has not yet occurred. Thus, depletion of Smu1/RED stalls spliceosome assembly after Prp28-mediated displacement of U1 (i.e. stable B complex formation), but before the first step of the activation process, namely, unwinding of the U4/U6 duplex by Brr2.

MS analysis indicated that U2, U5 and U4/U6 snRNP proteins are present in similar amounts (based on peptide counts) in the ΔSmu1/RED and kinetically-stalled B complexes (Supplementary Data 3). Only few Smu1 and RED peptides were detected in ΔSmu1/RED-B complexes, consistent with their efficient depletion, while the level of all other B-specific proteins remained essentially unchanged. Splicing factors that stably integrate during spliceosome activation—e.g. Prp19/CDC5L complex or B^act^ proteins—were underrepresented, consistent with the ΔSmu1/RED spliceosomes being stalled prior to activation. Thus, Smu1/RED depletion does not lead to major changes in spliceosome composition (other than loss of Smu1/RED), but potentially affects the structure of the B complex, as evidenced by its lower *S*-value.

### Physical separation of the 5′SS and BS restores activation

**Fig. 6:**
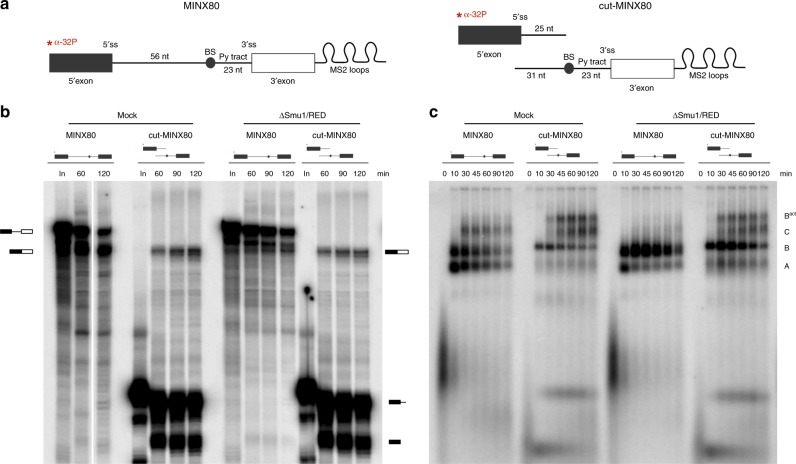
Cleavage of the MINX80 intron restores spliceosome activation in ΔSmu1/RED nuclear extract. **a** Schematic of the MINX80 and cut-MINX80 pre-mRNAs. Cut-MINX80 was generated by transcribing the MINX80 pre-mRNA in two parts, where only the 5′ half was ^32^P-radiolabelled (indicated in red). **b**, **c** Kinetics of in vitro splicing (**b**) and spliceosome assembly (**c**) of the MINX80 and cut-MINX80 pre-mRNAs in mock- and Smu1/RED-depleted extract. The pre-mRNA and splicing intermediates/products, as well as spliceosomal complex formation, were analysed as in Fig. 2. Source data are provided as a Source Data file

In contrast to long introns, which offer more flexibility, steric hindrance may arise from the physical constraints exerted by small introns, preventing spliceosomes assembled in the absence of Smu1/RED to adopt a conformation compatible with their subsequent activation. To test this idea, we released any potential physical constraints exerted by the short MINX80 intron by transcribing MINX80 in two independent parts (cut-MINX80) (Fig. 6a). The first comprised the 5′ exon plus 25 downstream intron nucleotides, and the second contained 31 nucleotides upstream of the BS, the BS, PPT and 3′ exon. As in this case only the 5′ exon is radiolabelled, the intron-3′ exon intermediate and spliced out intron are not detectable in in vitro splicing assays. In mock-depleted extract, splicing was less efficient with the cut versus intact MINX80, which is typical for *trans*-splicing (i.e. splicing of physically separated 5′ and 3′ substrates); there also appeared to be fewer A and B complexes formed, and an accumulation of B^act^/C complexes with the cut substrate (Fig. 6b, c). Whereas the splicing of intact MINX80 was much less efficient in ΔSmu1/RED extract compared to mock, similar levels of mRNA were formed in both extracts with the cut-MINX80 RNA (Fig. 6b). In addition, the strong accumulation of B complexes in ΔSmu1/RED extract was less severe with the cut-MINX80 (Fig. 6c). These results suggest that when the distance between the 5′SS and BS is short (i.e. ca 56 nts), physical constraints hinder the B to B^act^ transition, but even more so when Smu1 and RED are absent. This in turn suggests that Smu1 and RED promote the formation of a B complex conformation that is favourable for its activation and aid in relieving constraints that hinder activation when the 5′SS to BS distance is short.

### Smu1 and RED knockdown hinders B^act^ formation in HeLa cells

**Fig. 7:**
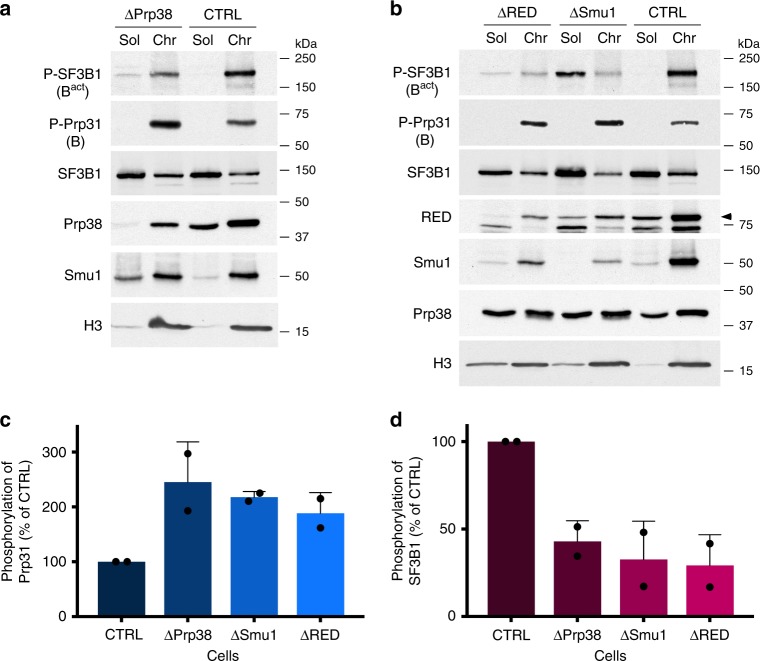
Smu1 and RED knockdowns alter the levels of phosphorylated Prp31 and SF3B1 in vivo. **a**, **b** Control HeLa cells (CTRL) or cells depleted of **a** Prp38 or **b** Smu1 and RED by RNAi were fractionated into a soluble (Sol) or chromatin (Chr) fraction. Equal amounts of each fraction were analysed by Western blot using antibodies against phosphorylated (P) SF3B1 or phosphorylated Prp31, SF3B1 (both phosphorylated and non-phosphorylated), Histone 3B (H3), RED, Smu1 or Prp38. **c**, **d** Quantification of phosphorylation levels of **c** Prp31 or **d** SF3B1 in CTRL or knockdown cells as indicated on the *X*-axis. The signal of the corresponding protein in the control lane was set to 100%. Error bars represent the standard deviation obtained from two independent experiments. The enhanced P-SF3B1 signal in the soluble nuclear fraction after Smu1 knockdown is likely due to dissociation during fractionation of less stable spliceosomes leading to leakage. Data points are shown as black dots. Error bars represent the standard deviation obtained from two independent experiments. Source data are provided as a Source Data file

To determine whether Smu1 and RED depletion also hinders spliceosome activation in vivo, we knocked down either Smu1, RED or, as a control, the B-specific Prp38 protein in HeLa cells by siRNA transfection. Cell nuclear extracts were fractionated into a soluble fraction and an insoluble fraction containing the chromatin-associated material. Phosphorylation of Prp31 occurs first during B complex formation^30^ and Prp31 is then released during activation, whereas hyperphosphorylation of SF3B1 is first observed in B^act^ complexes, and persists until step 2 of splicing^31,32^. Thus, antibodies directed against a phosphorylated form of Prp31 (P-Prp31) or SF3B1 (P-SF3B1) can be used to determine the relative amounts of B complexes or B^act^/C complexes, respectively, present in a given sample. P-Prp31 and P-SF3B1 were detected by Western blot primarily in the chromatin fraction (Fig. 7a, b), consistent with previous studies showing that approximately 80% of splicing events occur co-transcriptionally while the nascent transcript is still in the process of being transcribed by RNA polymerase II^31,33,34^. Human Prp38, like its yeast counterpart appears to be required for spliceosome activation^12^. In ΔPrp38 cells, the P-Prp31 signal in the chromatin fraction was stronger compared to that in control cells, whereas the chromatin-associated P-SF3B1 signal was reduced (Fig. 7a, c, d). This indicates that there is a block or slowdown in the B to B^act^ transition (i.e. spliceosome activation), as opposed to a general reduction in spliceosome assembly. A similar increase in the P-Prp31 signal and decrease in P-SF3B1 signal in the chromatin fraction was observed in ΔSmu1 and ΔRED cells (Fig. 7b–d), indicating a reduction in the amount/rate of transformation of B complexes into B^act^/C complexes in vivo upon Smu1 or RED knockdown. These data suggest that the splicing changes detected by RNA-Seq in Smu1- and RED-depleted cells might, at least in part, be due to a less efficient/slower spliceosome activation step.

## Discussion

Here, we investigated the function of the B-specific proteins Smu1 and RED in pre-mRNA splicing via a combination of in vivo and in vitro approaches. Our studies reveal that they not only play important roles during alternative splicing, but also function as general splicing factors, facilitating the activation of the human spliceosome. Several potential mechanisms exist whereby Smu1 and RED might affect spliceosome activation. Protein–protein crosslinking of purified human B complexes revealed that Smu1 and RED contact multiple proteins within the spliceosome^29^. Thus, Smu1 and RED were proposed to act as binding platforms for other spliceosomal proteins^20,21^ and their absence could potentially affect the recruitment of other proteins important for spliceosome activation. However, with the exception of the absence of Smu1/RED, the composition of ΔSmu1/RED B complexes was essentially identical to B complexes assembled in mock-depleted extract. Thus, the recruitment of B complex proteins is not dependent on Smu1/RED and the less efficient activation observed in their absence is not due to the absence of other proteins. However, we cannot rule out that depletion of Smu1 and RED alters protein–protein or protein–RNA interactions within the spliceosome. Indeed, ΔSmu1/RED B complexes exhibited an aberrant sedimentation behaviour on glycerol gradients, suggesting structural changes in the spliceosome, in particular a less compact conformation. Future cryo-EM of ΔSmu1/RED B complexes may allow insights into the nature of structural changes that may arise due to the absence of these proteins.

Spliceosome activation is initiated by Brr2-mediated unwinding of the U4/U6 duplex. As Brr2 is a stable component of the spliceosome prior to activation, its activity must be tightly regulated to prevent premature activation. Several mechanisms repress Brr2 activity prior to activation, and its RNA unwinding activity must be triggered, by a currently unknown mechanism, to start the activation process^35^. Smu1 and RED bridge Brr2 and the U2 protein SF3B3 in the B complex, with Smu1 directly contacting the N- and C-terminal helicase cassettes of Brr2. Thus, these proteins could help to position Brr2 relative to its substrate in a way that promotes U4/U6 unwinding. Consistent with this idea, Brr2 is very flexible and undergoes a large scale movement from its position in the tri-snRNP to its position close to U4/U6 in the B complex^29^, and Smu1 and RED could help to anchor Brr2 in this position. Enhanced Brr2 and/or U2 flexibility could also explain why B complexes lacking Smu1/RED appear to have a less compact structure. Furthermore, as Smu1’s WD40 domain is positioned at the interface of Brr2’s N- and C-terminal helicase cassettes, it could also potentially modulate Brr2’s helicase activity.

**Fig. 8:**
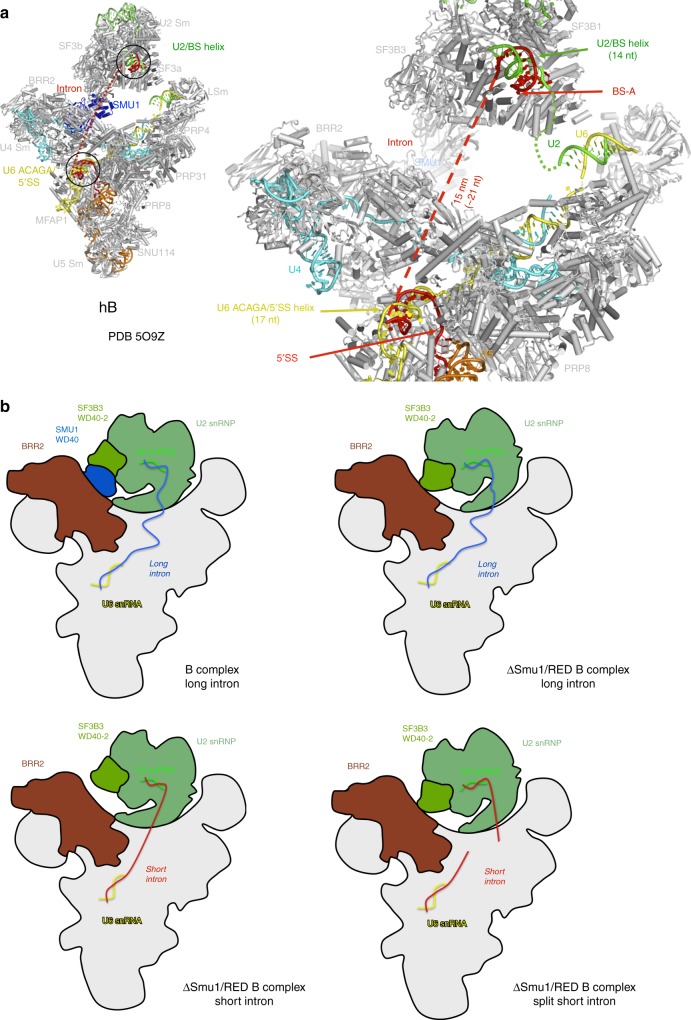
Model for how the 5′SS–BS distance leads to the dependence of spliceosome activation on Smu1/RED. **a** Distance between 5′SS and BS in the human B complex^29^. Left, overview of the complete human B complex. Right, expanded view of the upper region where intron nucleotides between the 5′SS and BS should be located in short introns. The intron (red solid line) forms extended helices with the U6 ACAGAG box and adjacent U6 nucleotides (yellow) at the 5′SS and the U2 snRNA (green) at the BS. The two helices are separated by 15 nm (ca 21 nt, red dashed line) in the human B complex. This would accommodate an estimated minimum of 52 nt between the 5′SS and the BS, without altering the structure of the spliceosome. **b** Model of the role of Smu1 and RED in the B complex. Upper left, Smu1/RED supports/stabilises the formation of the Brr2-U2 bridge, which is likely important for spliceosome activation. In the absence of Smu1/RED, this bridge can still be formed, albeit at a slower rate, as long as the distance between the 5′SS and BS is sufficiently long to allow movement of the U2 domain towards Brr2, resulting in a direct Brr2-U2 interaction. Lower left, when the distance between the 5′SS and the BS is short, the U2 snRNP may be repositioned and/or its ability to move towards Brr2 may be inhibited due to the structural constraint of the intron. Therefore, no direct contact between U2 and Brr2 can be established, and Smu1 and RED become essential for activation. Lower right, splitting the MINX80 pre-mRNA into two pieces may relieve this constraint, allowing the B-to-B^act^ transition even in the absence of Smu1 and RED. Panel (**a**) was reprinted from Bertram, K. et al. Cryo-EM structure of a pre-catalytic human spliceosome primed for activation. *Cell* **170**, 701–713 (2017), with permission from Elsevier

Introns retained after knockdown of Smu1 or RED were predominantly shorter than 200 nts. Nonetheless, a substantial fraction (30–40%) of the retained introns were significantly longer, consistent with a general requirement for Smu1 and RED for efficient splicing also of longer introns. In vitro splicing studies using truncated versions of MINX120 pre-mRNA, also demonstrated that spliceosome activation is more dependent on Smu1 and RED when intron length is shorter. Our data further indicate that the 5′SS–BS distance, rather than intron length per se, is a decisive factor for whether or not activation is strongly dependent on Smu1/RED. In the human B complex, a 17 nt long extended helix is formed between the U6 ACAGA box plus adjacent U6 nucleotides and intron nucleotides near the 5′SS^29^. Similarly, the BS and intron nucleotides upstream of it, form a 14 nt long helix with U2 snRNA^29,36,37^. In human B complexes formed on MINX120 pre-mRNA, these two extended helices are separated by 15 nm (Fig. 8a), which corresponds to ~21 nt of RNA in an extended conformation. Consequently, to span the distance between the 5′SS and BS, without altering the structure of the spliceosome, a minimum of ~52 intron nts would be necessary. This is approximately the distance found in the MINX80 and PM5-116 pre-mRNAs, suggesting that intron nucleotides between the extended U6/5′SS helix and U2/BS helices are likely in a fully-extended conformation in B complexes formed on these pre-mRNAs. This could limit the flexibility of the spliceosome during its remodelling, thereby impeding or—in the absence of Smu1/RED—completely preventing its activation. Consistent with this, physically separating the 5′SS and BS to release any length limitations, restored the transformation of B complexes into B^act^.

By combining the data presented here and information about the structure of the human B complex, we generated a model for how the 5′SS–BS distance might lead to the dependency of spliceosome activation on Smu1/RED (Fig. 8b). In the human B complex, Smu1 and RED form a molecular bridge between the U2 snRNP and tri-snRNP^29^. In the yeast spliceosome, which lacks Smu1 and RED, this bridge is formed directly by the Brr2 and SF3B3 homologs^38^ (Supplementary Fig. 6). Here, a WD40 domain of Rse1, the yeast counterpart of SF3B3, contacts the two helicase cassettes of Brr2, whereas in the human B complex the WD40 domain of Smu1 contacts them. This interaction likely stabilises the position of Brr2 relative to its U4/U6 substrate or might even directly stimulate Brr2 activity thereby promoting activation. In the human spliceosome, Smu1 and RED could ensure that the contact between U2 snRNP and Brr2 is established faster and remains stable during activation. As longer introns allow spliceosome activation in the absence of Smu1 and RED, but with reduced efficiency, it appears that a sufficiently long intron grants the U2-containing head domain of the B complex enough flexibility to move towards Brr2, coincidentally forming the apparently essential SF3B3-Brr2 bridge, only at a slower pace (Fig. 8b). In contrast, an intron with a critical 5′SS–BS distance of ~56 nt would be completely extended between those nucleotides forming a duplex with U2 or U6, and therefore physically restrict the mobility of U2. This would prevent the movements necessary for U2 SF3B3 to contact Brr2, and this would be most severe when Smu1 and RED are absent (Fig. 8b). In this case, physically separating 5′SS and BS would release this physical constraint. Consequently, the flexibility of U2 that allows it to move towards Brr2 would be restored, allowing spliceosome activation even without Smu1 and RED.

Previous studies, as well as data presented here, demonstrate that Smu1 and RED affect the outcomes of many alternative splicing events. Our data showing an effect of Smu1 and RED depletion on the rate of spliceosome activation and that introns with a short 5′SS–BS distance are especially dependent on Smu1/RED could explain, at least in some cases, how Smu1 and RED knockdown affects alternative splicing in vivo. For example, the use of an alternative upstream 5′SS would expand the 5′SS–BS distance of an alternatively spliced intron, and thus usage of a more distal 5′SS would be favoured after Smu1/RED knockdown in those cases where this distance is short. As alternative 3′SS usage often also involves the use of an alternative BS, the same may also be true when a downstream 3′SS is preferentially used in Smu1/RED-depleted cells. Transcription and splicing are temporally and functionally linked; splice site recognition by splicing factors and thus the assembly of a spliceosome at a particular splice site are affected by the pace of RNA Pol II elongation, which determines the rate at which splice sites and regulatory sequences appear in nascent pre-mRNAs (reviewed in ref. ^39^). In many cases, slowing the rate of RNA Pol II elongation favours the use of weaker upstream splice sites^40,41^. A slowed-down rate of spliceosome assembly and/or activation could enhance the use of weaker splice sites that are normally skipped in favour of stronger splice sites when the spliceosome assembly process is very fast. Thus, some changes in alternative splicing observed by RNA-seq analyses in Smu1- and RED-depleted cells might be due to a general slow-down in the formation of catalytically active spliceosomes. Although splice site selection is often determined prior to B complex formation, and thus prior to the recruitment of Smu1 and RED, some splice site choices appear to be made after A complex formation^42–44^. In addition, most spliceosome assembly steps are reversible^43,45^, and stalling at the B complex stage would likely also lead to enhanced dissociation and reformation of the spliceosome, which could also lead to altered splice site selection when Smu1 and RED are knocked down. Results from genome-wide screens and functional network reconstructions in multiple organisms indicate that changes in the activity of core splicing factors can influence alternative splicing^15,46–49^. Differential effects on splicing in other tissues may also arise due to differences in the expression levels of Smu1 and/or RED, where low levels of these proteins may become rate-limiting, leading to even more pronounced effects.

## Methods

### siRNA sequences

For RNA-Seq analysis, HeLa cells were transfected with siRNAs specific for human Smu1, RED, MFAP1 or Prp38 as described below.

RED: AACCCGTAACAAGAAGCTTAA; CAGCGAGTATATGAACAACAA; Smu1: CACAACTGAGCAGGCATATTA; TACGGCTATGTCGATCGAAAT; MFAP1: AAGGACCGAGTGACAGTTCAA; TACGGCGTTTACAGAACCGTA; Prp38: CTGCTGTTATTCAAAGATCAA; TCCACGGACATCGATAATCAA. A control siRNA with no target in the human transcriptome was also transfected. Cells were harvested 50 h post-siRNA transfection and their total RNA was extracted and reverse transcribed.

### RNA-Seq read alignment

Pair ended read libraries for the assayed condition were produced using the Trueseq v3 HiSeq sequencing kit. After de-multiplexing, the reads (pair-ended, 100 nt) were aligned against the human genome (version GRCh37/hg19) using tophat^50^ version 2.08 with command line parameters: --no-discordant --no-mixed --library-type fr-firststrand --mate-std-dev 85 -i 20 -I 800000 -x 5. In total ~900 million reads, or ca 225 million reads per condition, were aligned to the genome and the resulting bam files were used as input for the detection and quantification of alternative splicing and intron retention events.

### Quantification of alternative splicing events

Quantification of alternative splicing events was performed using a junction-centric approach that fuses available transcript structure information with experimental junction evidence. As our approach does not fully rely on existing splicing annotation it is capable of identifying novel splicing events that are absent in transcriptome databases. This is critical in experiments, such as those performed in this study, where drastic perturbations of the splicing machinery can result in a large number of novel/aberrant splicing events. *Junction reads* were defined as reads mapping discontinuously on the same gene, interrupted by a gene segment, containing canonical or non-canonical splice sites. The minimum overhang required for considering a junction read was set to 3 nucleotides for annotated junctions and 6 nucleotides for novel junctions. For each junction read a set of *neighbouring junction reads* were selected in order to calculate a normalised junction usage efficiency. We defined as a neighbouring junction only those for which all of the following conditions are met: (i) all known transcripts containing the neighbouring junction must fully subsume the original junction, (ii) all known transcripts containing the original junction must fully subsume the neighbouring junction and (iii) the neighbouring junction is within 5 kb of the original junction. *p*-Values for the differential usage of a junction in two conditions were calculated using Fisher’s exact test on the 2 × 2 contingency table containing the counts of a junction and its corresponding neighbouring junctions in the two conditions. The inclusion of all neighbouring junctions in the *p*-value calculation (as opposed to only junctions with common boundaries as is typical in PSI calculations, see below) reduces sensitivity to sampling noise and junction mappability differences in the RNA-Seq libraries. Percent Spliced In indices (PSI) were also calculated for each junction considering the neighbouring junctions with one common boundary (junction start or junction end) as:

1 $${\mathrm{PSI}} = {\mathrm{mean}}\left( \frac{J}{J_{{\mathrm{same}}\, {\mathrm{Start}}} +J},\frac{J}{J_{{\mathrm{same}} \, {\mathrm{End}}} + J} \right)$$

Differentially used junctions that can be attributed to alternative splicing were then selected on the basis of a series of thresholds applied to the previous metrics. For this study we required a *p*-value < 0.001, ΔPSI > 0.15 and at least 7 junction reads supporting the junction in one of the two conditions. Categorisation of the identified differential splicing junctions into different splicing competition types was performed by identifying competing junctions using a greedy local search around each differential junction. Competition types were classified as alternative 3′SS (Alt 3′SS), alternative 5′SS (Alt 5′SS), cassette exons (CEx) and complex events, such as those involving competition among multiple junctions on non-mutually exclusive junctions.

### Detection and quantification of intron retention

Detection of intron retention events relies on two lines of evidence that consider both junction and intronic reads: (i) The presence of a differentially used junction on the basis of the *p*-value threshold—see above—without a competing junction and (ii) differential intron retention (IR) for the intron in question on the basis of intronic read coverage. After normalising the number of intronic reads to the total number of intronic reads within an experiment, IR was calculated as the ratio of the strictly intronic reads covering a segment of the intron over the reads of neighbouring introns (as defined above) in order to account for intron read coverage shifts resulting from changes in gene expression. A singe robust IR value per intron was then calculated as the median of max (2, intron length/500) intronic segments IR values in order to reduce the number of false positives due to differential coverage in small portions of the intron. Such small spikes of intronic read coverage within larger introns can be products of intron-contained transcribed units such as unannotated exons, overlapping genes or autonomously transcribed transposable elements. A *p*-value for differential intron retention was finally calculated using Fisher’s exact test on the 2 × 2 contingency table containing the intronic counts for the segments of an intron in question and the corresponding neighbouring intronic reads from the two conditions. In addition to the *p*-value threshold for a differentially used junction (defined above) supporting intron retention we require at least a 2-fold change in IR and a p-value for differential intron retention <0.001 in order to consider an intron as differentially retained in two conditions. The code for the detection and quantification of alternative splicing events and the detection and quantification of intron retention is available as a single perl package in https://github.com/ppapasaikas/SANJUAN.

### Analysis of 5′SS–branch site (BS) distances

For the analysis of 5′SS–BS distances, BS data were obtained from the genome wide study by Pineda and Bradley^51^. The high and moderate confidence collection of human branch sites was overlapped with the collection of identified introns of this study. In cases where multiple BSs were linked to the same 5′SS of an intron, only the BS with the highest number of sequenced lariats with a mismatch at the BS nucleotide was considered. Of the ~80,000 introns that were confidently identified in this study, information about the BS was available for ~58% of them. This percentage was lower for shorter introns (e.g. 45% for introns <250 nt, 27% for introns <100 nt). Similar detection biases were also seen in other genome wide studies of human branch sites^52,53^. Relevant to this study, this detection asymmetry means that the observed bias for short 5′SS–BS distances in the pool of ΔSmu1/ΔRED retained introns is underestimated. Testing for differences in the distributions of 5′SS–BS distances between retained and unaffected introns was done using the two-sided, two-sample Smirnov–Kolmogorov test. For the unaffected introns, we used a sample of introns with matching length distributions to the retained introns with an oversampling factor of 5.

### Preparation of nuclear extract and Smu1/RED immunodepletion

For preparation of nuclear extract^52^ HeLa S3 cells (Helmholtz Center for Infection Research, Braunschweig) were pelleted by centrifugation in a Megafuge (Heraeus) at 2000 × *g* and 4 °C for 10 min. After two washes with 1× PBS (130 mM NaCl, 27 mM KCl, 8.0 mM Na_2_HPO_4_, 1.5 mM KH_2_PO_4_), cells were resuspended in 1.25 volumes MC Buffer (10 mM HEPES, pH 7.6, 10 mM KOAc, 0.5 mM Mg(OAc)_2_ and freshly added 5 mM dithiothreitol (DTT) and 1× cOmplete™, EDTA-free Protease Inhibitor Cocktail). Cells were incubated on ice for 5 min and dounced 18 times (Pestel B).

The extract was transferred to 50 mL falcon tubes and centrifuged in a F14-14 × 50cy rotor (Sorvall LYNX 6000 Superspeed Centrifuge) at 4 °C and 18,000 × *g* for 5 min. The pellet was resuspended in 1.3 volumes of Roeder C Buffer (25% glycerol, 20 mM HEPES-KOH, pH 7.9, 420 mM NaCl, 1.5 mM MgCl_2_, 0.2 mM EDTA-KOH, pH 8.0), and freshly added 0.5 mM dithioerythriol (DTE) and phenylmethanesulfonyl fluoride (PMSF) and dounced 20 times (Pestel B). The extract was kept in a beaker at 4 °C for 40 min and then transferred to a 50 mL falcon tube and centrifuged at 26,048 × *g* for 30 min at 4 °C in a F14-14 × 50cy rotor (Sorvall LYNX 6000 Superspeed Centrifuge). The nuclear extract (supernatant) was filled into fresh 50 mL falcon tubes and frozen in liquid nitrogen for storage at −80 °C or dialysed against Roeder D Buffer (20 mM HEPES-KOH, pH 7.9, 100 mM KCl, 1.5 mM MgCl_2_, 0.2 mM EDTA-KOH, pH 8.0) and freshly added 0.25 mM DTT and 0.25 mM PMSF. Immunodepletion of Smu1/RED from HeLa nuclear extract was performed using anti-peptide antibodies specific for Smu1, which were covalently crosslinked to Protein A Dynabeads (Thermo Fisher) using dimethyl pimelimidate (DMP). The anti-Smu1 beads were washed 3× with PBS and then blocked overnight in PBS supplemented with 0.5 mg/mL BSA, 0.05 mg/mL *Escherichia coli* tRNA and 0.05 mg/mL glycogen. The salt concentration of the nuclear extract was increased to 750 mM KCl. After three washes with Roeder D buffer containing 750 mM KCl (RD-750) (20 mM Hepes-KOH, pH 7.9, 750 mM KCl, 1.5 MgCl_2_, 0.2.mM EDTA, pH 8.0), 0.25 mM DTT, 0.25 mM PMSF, the beads were divided into two equal portions and the nuclear extract was incubated with each portion for 2 h at 4 °C with head-over-tail rotation. The nuclear extract was subsequently dialysed against RD-100 buffer for 5 h at 4 °C. Mock-depleted extract was treated in an identical manner, except the antibody was omitted.

### Purification of recombinant Smu1, RED or Smu1/RED dimer

Smu1 and RED were expressed separately or co-expressed in Sf9 (Thermo Fisher Scientific, Cat#11496015) or High Five (Thermo Fisher Scientific, Cat#B85502) cells using synthetic genes optimised for expression (GeneArt, Life Technologies). To purify the dimer, RED was tagged with an N-terminal poly-His_10_ tag and Smu1 was tagged with a C-terminal Strep-II tag. For the isolation of Smu1 alone, it was tagged with an N-terminal His_10_ tag (EcoRI/PstI), which allowed more efficient purification than the Strep-II tag. Plasmids encoding Smu1 or RED were transformed into DH10MultiBacY cells to generate baculoviral DNA. The latter was transfected in Sf9 insect cells grown in Gibco^®^ Sf-900™ III SFM medium, using X-tremeGENE™ 9 DNA Transfection Reagent according to the manufacturer protocol. 60–72 h after transfection, the virus containing supernatant was collected (V_0_). Fresh Sf9 cells were subsequently transfected to generate the V_1_ virus. For protein expression, the optimised High Five^TM^ cell line was used. The cells were maintained in suspension in ESF 921 Insect Cell Culture Medium and were harvested 60–72 h after virus transfection. The High Five^TM^ cell pellet was resuspended in pre-chilled High Five Lysis Buffer (50 mM Hepes-KOH, pH 7.9, 500 mM NaCl, 15 mM imidazole, 0.1% NP-40, 15% (v/v) glycerol, 2 mM DTT) (10 mL/g cells) and disrupted by ultrasonic probe sonication at 30% amplitude for 15–30 min (30 ms on, 30 ms off) until the lysate was clear. After centrifugation for 1 h at 17,000 × *g* in a F14-14 × 50cy rotor (Sorvall LYNX 6000 Superspeed Centrifuge) at 4 °C, the soluble cell fraction was diluted 1:1 with High Five Dilution Buffer (50 mM Hepes-KOH, pH 7.9, 50 mM NaCl, 15% (v/v) glycerol, 2 mM DTT) and incubated with previously equilibrated Roti^®^garose-His/Ni Beads (Roth) at 4 °C for 3 h with head-over-tail rotation. The beads were collected and washed 3× with High Five Wash Buffer (50 mM Hepes-KOH, pH 7.9, 750 mM NaCl, 20 mM imidazole, 15% (v/v) glycerol, 2 mM DTT) for 10 min at 4 °C with head-over-tail rotation. Elution was performed with 1.5–3 bead volumes of High Five Elution Buffer (20 mM Hepes-KOH, pH 7.9, 200 mM NaCl, 300 mM imidazole, 15% (v/v) glycerol, 2 mM DTT) on ice for 15 min. Purified proteins were analysed by SDS-PAGE, followed by staining with Coomassie blue, and the identity of the proteins was confirmed by mass spectrometry.

### In vitro splicing and splicing complex formation

Transcription templates for the MINX70, MINX80, MINX90 and MINX150 pre-mRNAs were generated by PCR using MINX120 as a template. PM5-211 was generated from the PM5 pre-mRNA (PM5-211) template in a similar manner. Templates for the 5′ and 3′ halves of MINX80 (cut-MINX80) were also generated by PCR. MINX80-extPy was obtained by duplicating a 10 nt stretch (TTTTTTTTCC) located between nucleotides 8 and 18 downstream of the BS and inserted after nucleotide 19. Uniformly ^32^P-labelled, m^7^G-capped pre-mRNA, or non-labelled 3′ half of cut-MINX80, was generated by in vitro transcription. Splicing reactions contained 40% (v/v) untreated or 50% (v/v) mock-depleted or Smu1/RED-depleted HeLa nuclear extract, 60 mM KCl, 3 mM MgCl_2_, 2 mM ATP 20 mM creatine phosphate, and 10 nM uniformly ^32^P-labelled, m^7^G-capped pre-mRNA, and were incubated at 30 °C for the indicated times. In the case of cut-MINX80, splicing was performed with 10 nM of the radiolabelled 5′ half and 50 nM of the unlabelled 3′ half. For splicing complementation experiments, a 30-fold molar excess of purified recombinant Smu1 or RED or Smu1/RED dimer was added to the Smu1/RED-depleted nuclear extract and preincubated for 15 min at 30 °C. Splicing was initiated by addition of the radiolabeled MINX-MS2 pre-mRNA. RNA was recovered at the indicated time points and separated on a 14% denaturing polyacrylamide gel. Unspliced pre-mRNA and spliced mRNA were detected using a Typhoon phosphoimager (GE Healthcare) and were quantified using ImageQuantTL (GE Healthcare). The percent spliced mRNA was calculated by dividing the amount of mRNA by the amount of pre-mRNA plus mRNA (minus background), and multiplying by 100. Spliceosomal complexes were analysed by electrophoresis in a 2% (w/v) low melting agarose gel in TBE buffer (89 mM Tris–HCl, pH 7.5, 89 mM boric acid, 2.5 mM EDTA, pH 8.0) in the presence of 0.5 μg/μl heparin, and bands were visualised with a Typhoon phosphoimager (GE Healthcare).

### MS2 affinity selection and mass spectrometry

For purification of spliceosomal complexes^53^, the pre-mRNA was pre-incubated with a 20-fold molar excess of purified MS2-MBP fusion protein and subsequently added to a 1 mL standard splicing reaction. Mock (kinetically-stalled) and ΔSmu1/RED B complexes were incubated for 8 min at 30 °C. The splicing reaction was loaded onto a 14 mL linear 10–30% (v/v) glycerol gradient containing G-150 buffer. Gradients were centrifuged at 60,000 × *g* for 15 h 20 min at 4 °C in a Sorvall TST 41.14 rotor, and 500 µl gradient fractions were harvested manually from the top. The distribution of ^32^P-labelled pre-mRNA was analysed by Cherenkov counting. For affinity selection, the peak fractions containing the respective spliceosomal complexes were pooled and loaded onto an amylose column (NEB). After washing with 50 column volumes of G-75 buffer (20 mM HEPES-KOH, pH 7.9, 1.5 mM MgCl_2_, 75 mM KCl), spliceosomal complexes were eluted by addition of G-75 buffer containing 25 mM maltose. The RNA composition was analysed by denaturing polyacrylamide-gel electrophoresis, followed by staining with SYBR^®^ Gold. Proteins were separated on 4–12% NuPAGE^TM^ gradient gels (Invitrogen), digested with trypsin, and peptides were analysed on an Q Exactive™ HF Hybrid Quadrupol-Orbitrap™ Mass Spectrometer (Thermo Fisher Scientific) under standard conditions. Proteins were identified by searching fragment spectra against UniProt (universal protein database) using Mascot as a search engine.

### siRNA-mediated knockdowns and cell fractionation

HeLa SS6 cells (ATCC, USA) were grown in 10 cm dishes containing Dulbecco’s Modified Eagle’s Medium (DMEM) supplemented with 10% fetal bovine serum (FBS) and 100 µg/mL penicillin/streptomycin. The latter was omitted prior to transfecting the cells with a control siRNA, or siRNAs against Smu1, RED or Prp38, which was performed using the Lipofectamine^®^ RNAiMAX Transfection Reagent (Thermo Fisher Scientific). 60 h after siRNA transfection, cells were washed twice with ice-cold 1× PBS and then HeLa Cell Lysis Buffer [2 mM EDTA, pH 8.0, 150 mM NaCl, 1 mM MgCl_2_, 30 mM Tris–HCl, pH 7.5, 1% (v/v) Triton X-100, 2× Complete Protease inhibitor (Roche), 2× PhosSTOP (Roche)] was added (~10 × 10^6^ cells/mL buffer). After brief vortexing, the samples were incubated on ice for 15 min. The nucleoplasmic fraction was separated from the chromatin fraction by centrifugation at 1100 × *g* for 10 min at 4 °C. The soluble fraction was collected and the pellet containing the chromatin fraction was resuspended in HeLa Cell Lysis Buffer in a volume equivalent to the soluble fraction. The chromatin was solubilized by sonication in a Bioruptor for 3 min (30 s on, 30 s off) at maximum intensity in a water bath at 2 °C. The protein concentration was determined using a BCA Protein Assay Kit (Thermo Fisher Scientific). The protein composition of the soluble and chromatin fractions was analysed by western blotting.

### Western blotting

For western blotting, proteins were separated by SDS-PAGE and transferred to a nitrocellulose membrane (Protran, Whatman). The blocked membrane was incubated with antibodies against the following human proteins Smu1 (1:1000; sc-100896—Santa Cruz Biotechnology, USA), Histone H3B (1:5000; ab18521—Abcam, UK), RED (1:1000; Lührmann Laboratory), FBP21 (1:1000; sc-84249 Santa Cruz Biotechnology, USA), Prp38 (1:3000; Lührmann Laboratory)^12^, MFAP1 (1:1000; Lührmann Laboratory), Snu114 (1:1500; Lührmann Laboratory)^54^, SF3A2 (1:2000; Lührmann Laboratory) and SF3B1 (1:1000; Lührmann Laboratory)^55^, Snu66 (1:2000; Lührmann Laboratory)^56^, phosphorylated SF3B1 (1:1500; Lührmann Laboratory)^31^, Prp31 (1:1000; Lührmann Laboratory)^57^, phosphorylated Prp31 (1:2000; Lührmann Laboratory)^30^. As secondary antibody horseradish-peroxidase-conjugated Goat-anti-rabbit (1:50,000; 111-035-144—Jackson Immunoresearch, USA) or Goat-anti-mouse (1:10,000; 115-035-003—Jackson Immunoresearch, USA) antibodies were used and bound antibody was detected using an ECL detection kit (GE Healthcare).

### Reporting summary

Further information on research design is available in the Nature Research Reporting Summary linked to this article.

## Supplementary Information

Supplementary information

Peer Review

Reporting summary

Description of Additional Supplementary Files

Supplementary Data 1

Supplementary Data 2

Supplementary Data 3

Source Data

## Data Availability

A reporting summary for this Article is available as a Supplementary Information file. The source data underlying Figs. 2b–d, 3b–d, 4a, b, d–f, 5b, 6b, c, 7a–d, and Supplementary Figs. 1a, b, 2a–c, 3a, 4b, c, 5b–e, as well as Supplementary Data 3, are provided as a Source Data file. Data that support the findings of this study have been deposited in the ebi ArrayExpress Archive of Functional Genomics Data repository. Accession code E-MTAB-7522. All data is available from the corresponding author upon reasonable request.

## Code availability

The code used for the detection and quantification of alternative splicing events and intron retention is available as a single perl package in https://github.com/ppapasaikas/SANJUAN.

## References

[1] Wahl MC, Will CL, Lührmann R (2009). The spliceosome: design principles of a dynamic RNP machine. Cell.

[2] Will, C. L. & Lührmann, R. Spliceosome structure and function. *Cold Spring Harb. Perspect. Biol.* **3**, a003707 (2011). 10.1101/cshperspect.a003707 PMC3119917 21441581

[3] Hube F, Francastel C (2015). Mammalian introns: when the junk generates molecular diversity. Int. J. Mol. Sci..

[4] Ruskin B, Greene JM, Green MR (1985). Cryptic branch point activation allows accurate in vitro splicing of human beta-globin intron mutants. Cell.

[5] Wieringa B, Hofer E, Weissmann C (1984). A minimal intron length but no specific internal sequence is required for splicing the large rabbit beta-globin intron. Cell.

[6] Fu XD, Katz RA, Skalka AM, Leis J (1988). Site-directed mutagenesis of the avian retrovirus nucleocapsid protein, pp 12. Mutation which affects RNA binding in vitro blocks viral replication. J. Biol. Chem..

[7] Himmelspach M, Gattoni R, Gerst C, Chebli K, Stevenin J (1991). Differential block of U small nuclear ribonucleoprotein particle interactions during in vitro splicing of adenovirus E1A transcripts containing abnormally short introns. Mol. Cell. Biol..

[8] Kohrer K, Domdey H (1988). Splicing and spliceosome formation of the yeast MATa1 transcript require a minimum distance from the 5′ splice site to the internal branch acceptor site. Nucleic Acids Res..

[9] Smith CW, Nadal-Ginard B (1989). Mutually exclusive splicing of alpha-tropomyosin exons enforced by an unusual lariat branch point location: implications for constitutive splicing. Cell.

[10] Agafonov DE (2011). Semiquantitative proteomic analysis of the human spliceosome via a novel two-dimensional gel electrophoresis method. Mol. Cell. Biol..

[11] Xie J, Beickman K, Otte E, Rymond BC (1998). Progression through the spliceosome cycle requires Prp38p function for U4/U6 snRNA dissociation. EMBO J..

[12] Schutze T (2016). Multiple protein–protein interactions converging on the Prp38 protein during activation of the human spliceosome. RNA.

[13] Chung T, Wang D, Kim CS, Yadegari R, Larkins BA (2009). Plant SMU-1 and SMU-2 homologues regulate pre-mRNA splicing and multiple aspects of development. Plant Physiol..

[14] Kanno T, Lin WD, Fu JL, Matzke AJM, Matzke M (2017). A genetic screen implicates a CWC16/Yju2/CCDC130 protein and SMU1 in alternative splicing in *Arabidopsis thaliana*. RNA.

[15] Papasaikas P, Tejedor JR, Vigevani L, Valcarcel J (2015). Functional splicing network reveals extensive regulatory potential of the core spliceosomal machinery. Mol. Cell.

[16] Spartz AK, Herman RK, Shaw JE (2004). SMU-2 and SMU-1, *Caenorhabditis elegans* homologs of mammalian spliceosome-associated proteins RED and fSAP57, work together to affect splice site choice. Mol. Cell. Biol..

[17] Spike CA, Shaw JE, Herman RK (2001). Analysis of smu-1, a gene that regulates the alternative splicing of unc-52 pre-mRNA in *Caenorhabditis elegans*. Mol. Cell. Biol..

[18] Sugaya K, Hongo E, Ishihara Y, Tsuji H (2006). The conserved role of Smu1 in splicing is characterized in its mammalian temperature-sensitive mutant. J. Cell Sci..

[19] Fournier G (2014). Recruitment of RED–SMU1 complex by Influenza A virus RNA polymerase to control viral mRNA splicing. PLoS Pathog..

[20] Hegele A (2012). Dynamic protein–protein interaction wiring of the human spliceosome. Mol. Cell.

[21] Ulrich AKC, Schulz JF, Kamprad A, Schutze T, Wahl MC (2016). Structural basis for the functional coupling of the alternative splicing factors Smu1 and RED. Structure.

[22] Neumann B (2010). Phenotypic profiling of the human genome by time-lapse microscopy reveals cell division genes. Nature.

[23] Rines DR (2008). Whole genome functional analysis identifies novel components required for mitotic spindle integrity in human cells. Genome Biol..

[24] Paulsen RD (2009). A genome-wide siRNA screen reveals diverse cellular processes and pathways that mediate genome stability. Mol. Cell.

[25] Ren L (2013). Loss of Smu1 function de-represses DNA replication and over-activates ATR-dependent replication checkpoint. Biochem. Biophys. Res. Commun..

[26] Sugaya K, Hongo E, Tsuji H (2005). A temperature-sensitive mutation in the WD repeat-containing protein Smu1 is related to maintenance of chromosome integrity. Exp. Cell Res..

[27] Lee S, Han S, Jeong AL, Park JS, Yang Y (2014). Depletion of IK causes mitotic arrest through aberrant regulation of mitotic kinases and phosphatases. FEBS Lett..

[28] Yeh PC, Yeh CC, Chen YC, Juang YL (2012). RED, a spindle pole-associated protein, is required for kinetochore localization of MAD1, mitotic progression, and activation of the spindle assembly checkpoint. J. Biol. Chem..

[29] Bertram K (2017). Cryo-EM structure of a pre-catalytic human spliceosome primed for activation. Cell.

[30] Schneider M (2010). Human PRP4 kinase is required for stable tri-snRNP association during spliceosomal B complex formation. Nat. Struct. Mol. Biol..

[31] Girard C (2012). Post-transcriptional spliceosomes are retained in nuclear speckles until splicing completion. Nat. Commun..

[32] Shi Y, Reddy B, Manley JL (2006). PP1/PP2A phosphatases are required for the second step of pre-mRNA splicing and target specific snRNP proteins. Mol. Cell.

[33] Khodor YL (2011). Nascent-seq indicates widespread cotranscriptional pre-mRNA splicing in Drosophila. Genes Dev..

[34] Pandya-Jones A, Black DL (2009). Co-transcriptional splicing of constitutive and alternative exons. RNA.

[35] Absmeier E, Santos KF, Wahl MC (2016). Functions and regulation of the Brr2 RNA helicase during splicing. Cell Cycle.

[36] Haselbach D (2018). Structure and conformational dynamics of the human spliceosomal B(act) complex. Cell.

[37] Zhang X (2018). Structure of the human activated spliceosome in three conformational states. Cell Res..

[38] Plaschka C, Lin PC, Nagai K (2017). Structure of a pre-catalytic spliceosome. Nature.

[39] Kornblihtt AR (2013). Alternative splicing: a pivotal step between eukaryotic transcription and translation. Nat. Rev. Mol. Cell Biol..

[40] de la Mata M (2003). A slow RNA polymerase II affects alternative splicing in vivo. Mol. Cell.

[41] Kadener S (2001). Antagonistic effects of T-Ag and VP16 reveal a role for RNA pol II elongation on alternative splicing. EMBO J..

[42] Bonnal S (2008). RBM5/Luca-15/H37 regulates Fas alternative splice site pairing after exon definition. Mol. Cell.

[43] Hoskins, A. A., Rodgers, M. L., Friedman, L. J., Gelles, J. & Moore M. J. Single molecule analysis reveals reversible and irreversible steps during spliceosome activation. *eLife* **5**, e14166 (2016). 10.7554/eLife.14166 PMC4922858 27244240

[44] House AE, Lynch KW (2006). An exonic splicing silencer represses spliceosome assembly after ATP-dependent exon recognition. Nat. Struct. Mol. Biol..

[45] Tseng CK, Cheng SC (2008). Both catalytic steps of nuclear pre-mRNA splicing are reversible. Science.

[46] Clark TA, Sugnet CW, Ares M (2002). Genomewide analysis of mRNA processing in yeast using splicing-specific microarrays. Science.

[47] Park JW, Graveley BR (2005). Use of RNA interference to dissect the roles of trans-acting factors in alternative pre-mRNA splicing. Methods.

[48] Pleiss JA, Whitworth GB, Bergkessel M, Guthrie C (2007). Transcript specificity in yeast pre-mRNA splicing revealed by mutations in core spliceosomal components. PLoS Biol..

[49] Tejedor JR, Papasaikas P, Valcarcel J (2015). Genome-wide identification of Fas/CD95 alternative splicing regulators reveals links with iron homeostasis. Mol. Cell.

[50] Trapnell C (2012). Differential gene and transcript expression analysis of RNA-seq experiments with TopHat and Cufflinks. Nat. Protoc..

[51] Pineda JMB, Bradley RK (2018). Most human introns are recognized via multiple and tissue-specific branchpoints. Genes Dev..

[52] Dignam JD, Martin PL, Shastry BS, Roeder RG (1983). Eukaryotic gene transcription with purified components. Methods Enzymol..

[53] Bessonov S (2010). Characterization of purified human Bact spliceosomal complexes reveals compositional and morphological changes during spliceosome activation and first step catalysis. RNA.

[54] Fabrizio P, Laggerbauer B, Lauber J, Lane WS, Lührmann R (1997). An evolutionarily conserved U5 snRNP-specific protein is a GTP-binding factor closely related to the ribosomal translocase EF-2. EMBO J..

[55] Will CL (2002). Characterization of novel SF3b and 17S U2 snRNP proteins, including a human Prp5p homologue and an SF3b DEAD-box protein. EMBO J..

[56] Makarova OV, Makarov EM, Lührmann R (2001). The 65 and 110 kDa SR-related proteins of the U4/U6.U5 tri-snRNP are essential for the assembly of mature spliceosomes. EMBO J..

[57] Makarova OV, Makarov EM, Liu S, Vornlocher HP, Lührmann R (2002). Protein 61K, encoded by a gene (PRPF31) linked to autosomal dominant retinitis pigmentosa, is required for U4/U6*U5 tri-snRNP formation and pre-mRNA splicing. EMBO J..

