# Supplementary material for: Smu1 and RED are required for activation of spliceosomal B complexes assembled on short introns
Source: Nat Commun. 2019 Aug 13;10:3639. doi: 10.1038/s41467-019-11293-8 (PMC6692369; doi:10.1038/s41467-019-11293-8)
Supplement: Supplementary file 1 — Supplementary information [file 41467_2019_11293_MOESM1_ESM.pdf]

Smu1 and RED are required for activation of spliceosomal B complexes assembled on short introns

Keiper *et al.*

Supplementary Figure 1

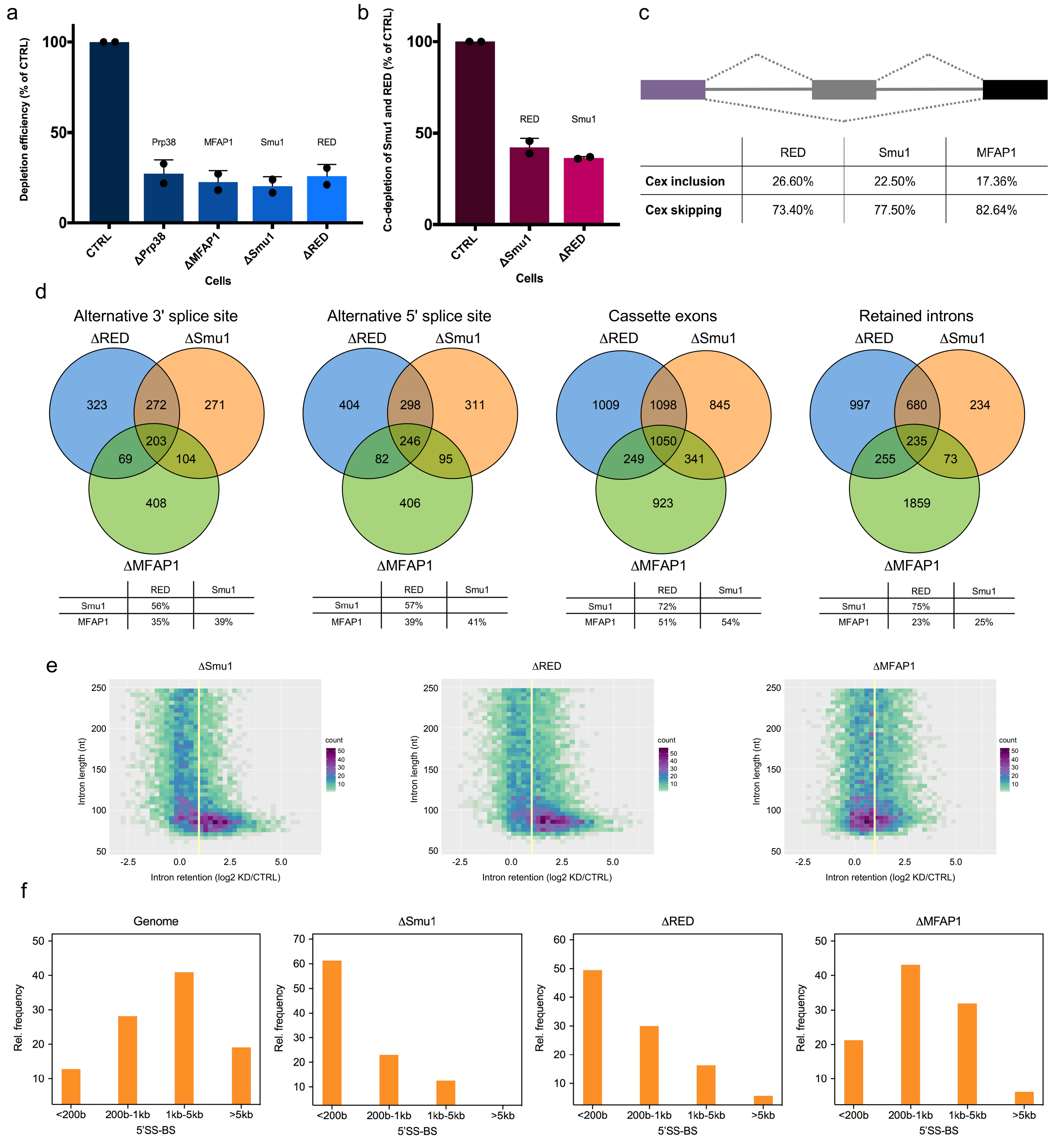

	RED	Smu1	MFAP1
**Cex inclusion**	26.60%	22.50%	17.36%
**Cex skipping**	73.40%	77.50%	82.64%

Alternative 3' splice site:

	RED	Smu1
Smu1	56%	
MFAP1	35%	39%

Alternative 5' splice site:

	RED	Smu1
Smu1	57%	
MFAP1	39%	41%

Cassette exons:

	RED	Smu1
Smu1	72%	
MFAP1	51%	54%

Retained introns:

	RED	Smu1
Smu1	75%	
MFAP1	23%	25%

Supplementary Figure 1 Smu1 and RED knockdown lead to changes in alternative splicing patterns. a,b Quantification of the depletion efficiency of the proteins shown above the bars in CTRL or knockdown cells as indicated on the X-axis. The signal of the corresponding protein in the control lane was set to 100%. Data points are shown as black dots. Error bars represent the standard deviation obtained from two independent experiments. c, Relative fraction of cassette exon inclusion or skipping among those cassette exons whose alternative splicing was affected in ΔSmu1, Δ RED or ΔMFAP1 HeLa cells. d, Venn diagrams showing the extent of overlap of AS events - i.e. usage of alternative 3' splice sites, usage of alternative 5' splice sites, and cassette exon skipping/inclusion, and retained introns that were altered after RNAi-mediated knockdown of Smu1, RED or MFAP1 in HeLa cells, as determined by RNA-seq. Numbers shown in the circles indicate the absolute number of AS events affected by the indicated knockdown. The table shows the relative overlap in %. e, Heat map showing the level of intron retention (x-axis) relative to the intron length (y-axis). The color code ranges from pale green for low abundant introns, to dark purple for highly abundant introns. The yellow line is set at log2 KD/CTRL=1. f, The distances between the 5'SS and BS-A were sorted into 4 classes according to their length - i.e. <200 bases, 200 to 1Kb, 1Kb to 5Kb and >5Kb. Charts showing the relative distribution of 5'SS-BS distances in the genome or in those introns retained after Smu1, RED or MFAP1 knockdown. Error bars represent the standard deviation obtained from two independent experiments.

Supplementary Figure 2

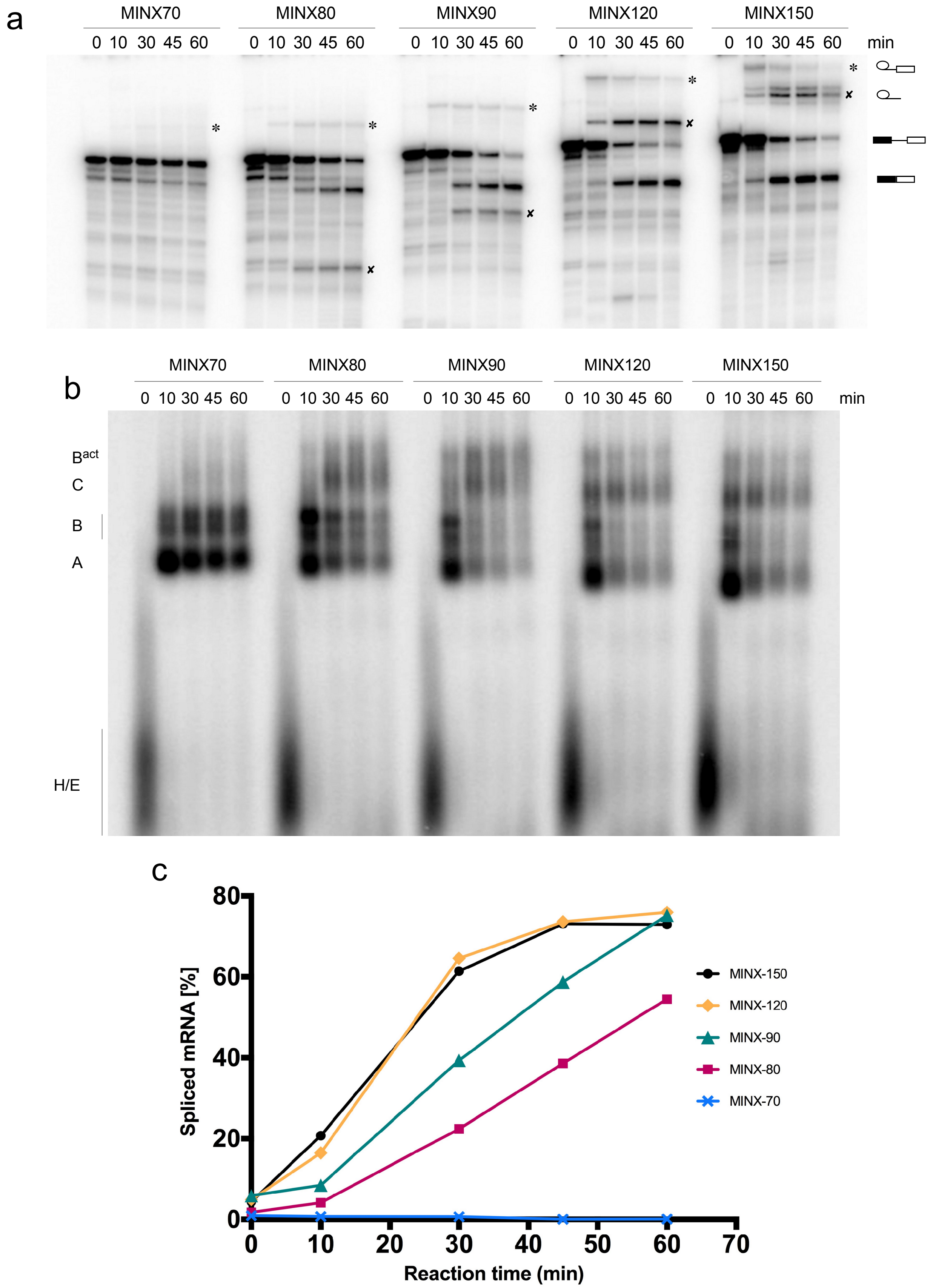

Supplementary Figure 2 Shortening the MINX pre-mRNA intron affects spliceosome assembly and splicing. a-b, Kinetics of in vitro splicing (a) and spliceosome assembly (b) of the indicated MINX pre-mRNA constructs (see Figure 2 for details). ^{32}P-labelled pre-mRNAs were incubated under splicing conditions in the presence of untreated HeLa nuclear extract for the indicated times. In panel a, RNA was analysed by denaturing PAGE and visualized by autoradiography. The pre-mRNA and splicing intermediates and products are indicated on the left. The asterisk and “x” indicate the intron-3'exon intermediate or spliced-out intron of the various pre-mRNAs, which migrate differently due to their varying sizes. In panel b, spliceosomal complex formation was analysed on a native agarose gel and visualized by autoradiography. The positions of the H/E, A, B, C, and B^{act} complexes are indicated on the left. c, Quantification of the percent of spliced mRNA formed at different time points with the indicated MINX pre-mRNAs. Source data are provided as a Source Data file.

Supplementary Figure 3

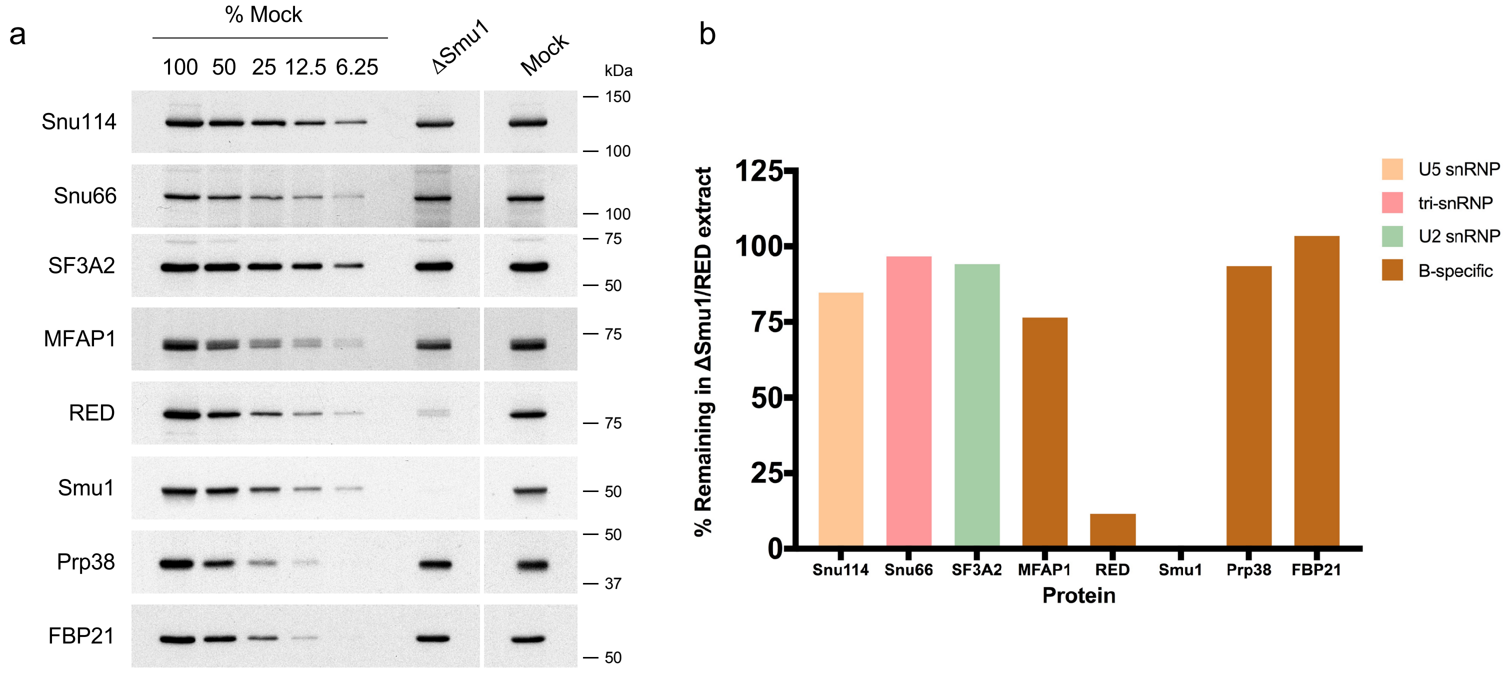

Supplementary Figure 3 Smu1 and RED are efficiently removed from HeLa nuclear extract by immunodepletion. a, HeLa nuclear extract was mock-depleted or immunodepleted using anti-Smu1 antibodies. Depletion efficiency was determined via immunoblotting, by comparing equal amounts (protein concentrations) of the mock-depleted or ΔSmu1 extract (set to 100%), and a dilution series (100 to 6.25%) of the mock-depleted extract. Proteins were visualized by immunoblotting using antibodies against Smu1 or RED, as well as core splicing factors (Snu114, Snu66 and SF3A2) or other B specific proteins (MFAP1, FBP21 and Prp38) as controls. b, Quantification of the amount of the indicated protein remaining after immunodepletion with anti-Smu1 antibodies.

Supplementary Figure 4

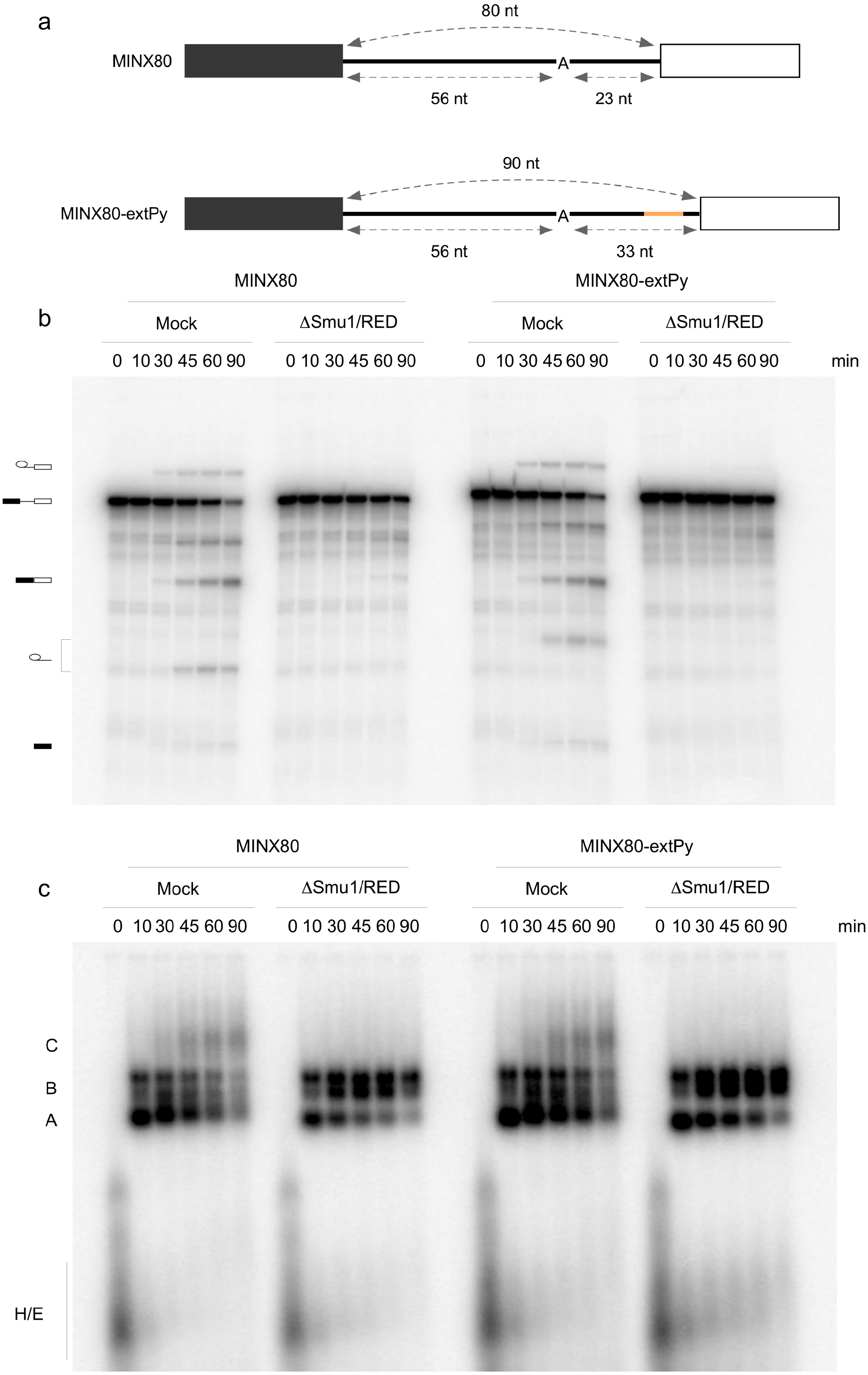

Supplementary Figure 4 Expanding the distance between the BS and 3'SS of MINX-80 does not relieve the block in spliceosome activation. a, Schematic of MINX80 and MINX80-extPy pre-mRNAs. By duplicating a stretch of the polypyrimidine tract of MINX-80, ten additional nucleotides were introduced between the BS and the 3'SS, generating MINX-80-extPY. The variable region is illustrated in orange. b-c, Kinetics of in vitro splicing (b) and spliceosome assembly (c) with MINX-80 versus MINX-80-extPY in the presence or absence of Smu1/RED. ^{32}P-labelled pre-mRNA was incubated under splicing conditions in the presence of mock-depleted (i.e., treated in an identical manner but without antibody) or Smu1/RED-immunodepleted HeLa nuclear extract for 0-90 min. In panel b, RNA was analysed by denaturing PAGE and visualized by autoradiography. The pre-mRNA and splicing intermediates and products are indicated on the left. In panel c, spliceosomal complex formation was analysed on a native agarose gel and visualized by autoradiography. The positions of the H/E, A, B, and C complexes are indicated on the left. Source data are provided as a Source Data file.

Supplementary Figure 5

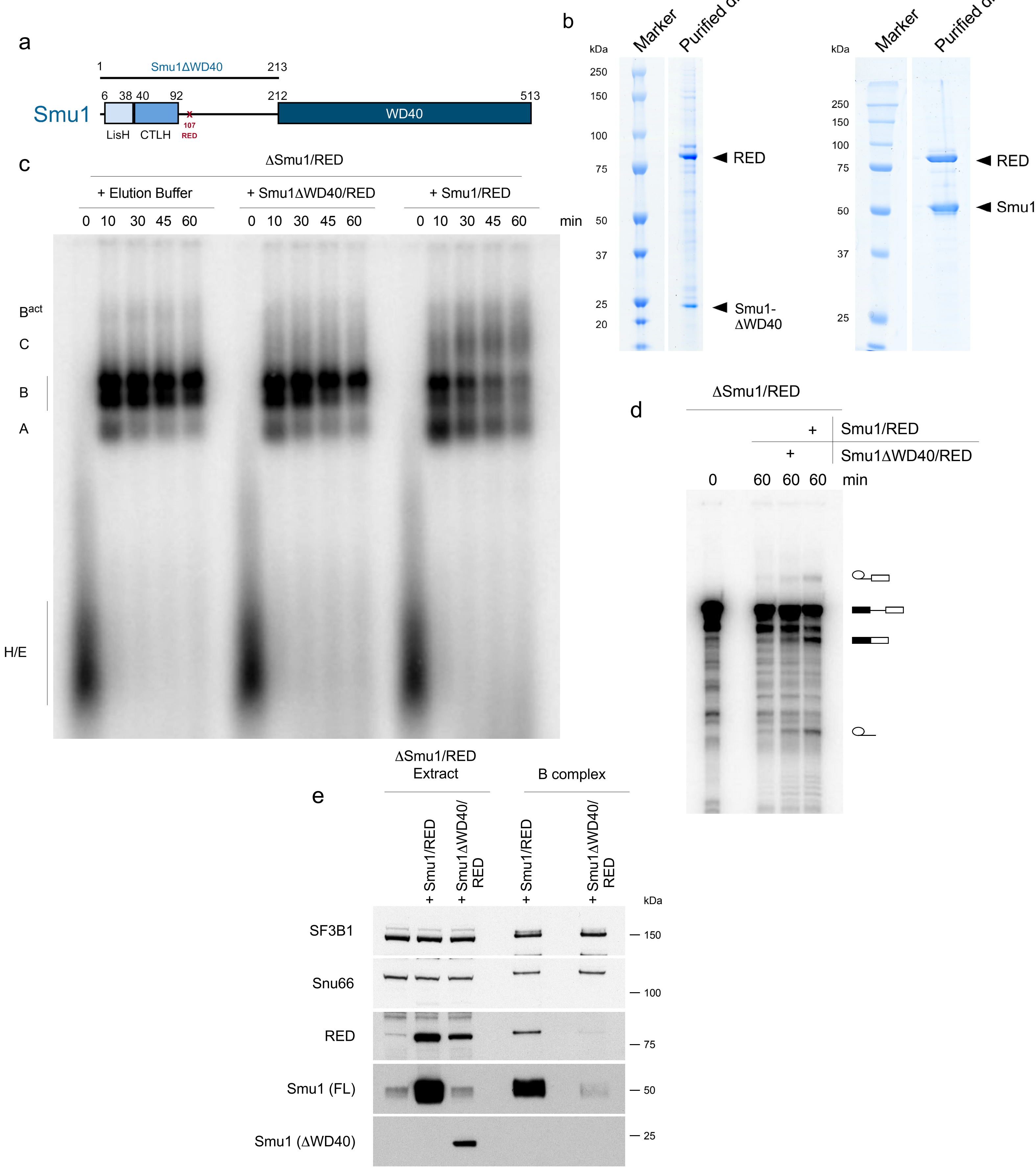

Supplementary Figure 5 Deletion of the WD40 domain of Smu1 prevents Smu1/RED from associating with the spliceosome. a, Schematic representation of the domain architecture of the Smu1 protein. Structural domains are indicated by blue boxes. LisH, lissencephaly type 1 homology motif, and CTLH, C-terminal to LisH motif. The amino acid (107) that was crosslinked to RED in the human B complex [29] is marked by a red "X" and indicates a Smu1/RED interaction region. The region of Smu1 (aa 1-213) that is present in the Smu1-ΔWD40 mutant is indicated above. b, Smu1 or Smu1-ΔWD40 was co-expressed with RED in insect cells and the Smu1/RED (right) and Smu1-ΔWD40/RED (left) dimer was affinity purified and analysed by SDS-PAGE. c-d, In contrast to the Smu1/RED dimer, neither the assembly of spliceosomal complexes on MINX80 pre-mRNA (c) nor splicing (d) were substantially restored after addition of purified Smu1-ΔWD40/RED dimer to Smu1/RED HeLa nuclear extract. The pre-mRNA and splicing intermediates and products, as well as spliceosomal complex formation were analysed as in Supplementary Figure 2. e, The Smu1-ΔWD40/RED dimer is not incorporated into spliceosomal B complexes. Smu1/RED HeLa nuclear extract or purified B complexes formed in Smu1/RED HeLa nuclear extract after addition of purified Smu1/RED or Smu1-Δ WD40/RED dimers (as indicated above) were analysed by Western blot. The presence of SF3B1 and Snu66 (which served as loading controls), or Smu1, Smu1-WD40, or RED was determined using antibodies against the proteins indicated on the left. Source data are provided as a Source Data file.

Supplementary Figure 6

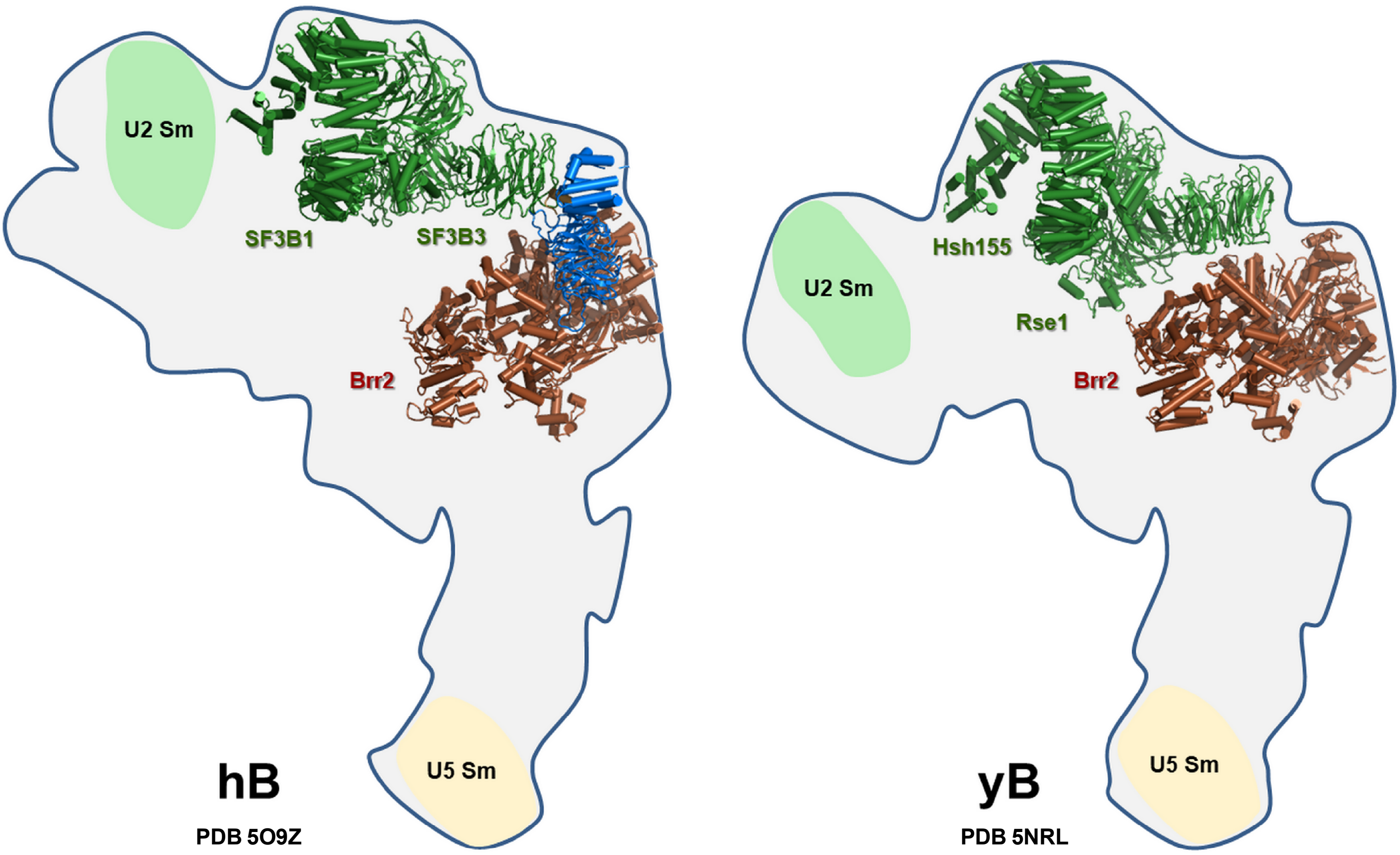

Supplementary Figure 6 Comparison of the structural organization of SF3B proteins and Brr2 in the human and S. cerevisiae spliceosomal B complex. The structure and location of the SF3B1 (yeast Hsh155) HEAT domain, and SF3B3 (yeast Rse1) WD40 domains (both proteins shown in green), and of Smu1/RED (colored blue and purple) and Brr2 (brown) shown in an outline of the human (left) [29] or S. cerevisiae (right) [38] B complex. For orientation, the U2 and U5 Sm cores are shown schematically. The human B complex was reprinted from Cell 170, Karl Bertram, Dmitry E. Agafonov, Olexandr Dybkov, David Haselbach, Majety N. Leelaram, Cindy L. Will, Henning Urlaub, Berthold Kastner, Reinhard Lührmann and Holger Stark, Cryo-EM Structure of a Pre-catalytic Human Spliceosome Primed for Activation, 701–713, 2017 with permission from Elsevier. The yeast B complex was reprinted from Nature volume 546, Plaschka C., Lin P. C., Nagai K., Structure of a pre-catalytic spliceosome, 617–621, 2017 with permission from Springer Nature Publishing AG.

Primer List

**Name**	**Sequence (5' → 3')**
Smu1-F	CAATCACTCGACGAAGAC
Smu1-R	CTTCCGTGTTTCAGTTAGC
Strep-D-WD40-R	ATTATGCATTCATTATTTTTCGAACTGAGGG
	TGGGACCACTGTCCGAACTTGATGTGCC
Smu1-His-F	ATACGAATTCCATATGTCTATCGAAATCGAGTCC
Smu1-His-R	ATACCTGCAGGATTTAAGGCTTCCACAGCTTCAGCA
RED-F	ATGCGAATTCAGCATGGGACCTGAAAGG
RED-R	ATGCCTGCAGGATGCTCATTAGTACTTAG
MINX-70 Primer A	TCACACAGGAAACAGCTATGAC
MINX-70 Primer B	TCTTACCGTTCGGAGG
MINX-70 Primer C	CCTCCGAACGGTAAGAGGGCGCAGTAGTCCAG
MINX-70 Primer D	GTAAAACGACGGCCAGTG
MINX-80 Primer A	TCACACAGGAAACAGCTATGAC
MINX-80 Primer B	AGTTCTACATGCTAGGCTCTTACC
MINX-80 Primer C	GGTAAGAGCCTAGCATGTAGAACT GTAGTCCAGGGTTTCCTT
MINX-80 Primer D	GTAAAACGACGGCCAGTG
MINX-90 Primer A	TCACACAGGAAACAGCTATGAC
MINX-90 Primer B	AGTTCTACATGCTAGGCTCTTACC
MINX-90 Primer C	GGTAAGAGCCTAGCATGTAGAACT CTAGGGCGCAGTAGTCCAG
MINX-90 Primer D	GTAAAACGACGGCCAGTG
MINX-150 Primer A	TCACACAGGAAACAGCTATGAC
MINX-150 Primer B	AGACTGAGACTGAGACTGAGGGCTCTTACCGTTCGGAG
MINX-150 Primer C	CTCAGTCTCAGTCTCAGTCTCAGTCTGCCTgtagaactggttacctgcagcc
MINX-150 Primer D	GTAAAACGACGGCCAGTG
MINX-80-cleaved Primer A	TCACACAGGAAACAGCTATGAC
MINX-80-cleaved Primer B	TGAGACTGAGACTGAGGGCTC
MINX-80-cleaved Primer C	TAATACGACTCACTATAGGGTCTCAGGGTTTCCTTGATG
MINX-80-cleaved Primer D	GTAAAACGACGGCCAGTG
MINX80-PyExtentionFor	ATAGGGAGACGGAATTCGAGCTCGCCCACTCTTGGATCGGAAACCCGTCG
	GCCTCCGAACGGTAAGAGCCTAGCATGTAGAACTGTAGTCCAGGGTTTCCT
	TGATGATGTCATACTTATC
MINX80-PyExtensionRev	TGGTGTACGGATATTGGATCCCCACTGGAAAGACCGCGAAGAGTTTGTCCT
	CAACCGCGAGCTGTGGAAAAAAAAGGAAAAAAAAGGGACAGGATAAGTATG
	ACATCATCAAGGAAACCC
MINX80-PyExtension-T7For	GGTACCTAATACGA CTCACTATAGGGAGACGGAATTCGAGC
MINX80-PyExtensionRev	TGTACGGATATTGGATCCCC

$PM5_{56}$ and $PM5\text{-}10_{56}$ Primer A	TCACACAGGAAACAGCTATGAC
$PM5_{56}$ and $PM5\text{-}10_{56}$ Primer B	GTAAGCTTGATACATACCTTGGC
$PM5_{56}$ and $PM5\text{-}10_{56}$ Primer C	GCCAAGGTATGTATCAAGCTTACGTGACTGATAGAACACTACCTG
$PM5_{56}$ and $PM5\text{-}10_{56}$ Primer D	GTAAAACGACGGCCAGTG

U1 snRNA forward K107	GATACTTACCTGGCAGGGGAG
U1 snRNA reverse K50	CGCGGATCCAGGGGAAAGCGCGAACGCAGTC
U2 snRNA forward K78	CCTAATACTCACTATAGATCGCTTCTCGGCCTTTTGCG
U2 snRNA reverse K79	GGGTGCACCGTTCCTGGAGGTAC
U4 snRNA forward K47	GGGAATTCCTAATACGACTCACTA
U4 snRNA reverse K48	CGCGAATCCAGTCTCCGTAGAGAC
U5 snRNA forward K115	GATACTCTGGTTTCTCTTCAG
U5 snRNA reverse KJ7	CCCAAGCTTTAGCCTTGCCAAGGCAAGG
U6 snRNA forward K54	CCTAATACGACTCACTATAGGTGCTCGCTTCGGCAGC
U6 snRNA reverse K55	AAAAATATGGAACGCTTCACG
