# Supplementary material for: Smu1 and RED are required for activation of spliceosomal B complexes assembled on short introns
Source: Nat Commun. 2019 Aug 13;10:3639. doi: 10.1038/s41467-019-11293-8 (PMC6692369; doi:10.1038/s41467-019-11293-8)
Supplement: Supplementary file 4 — Description of Additional Supplementary Files [file 41467_2019_11293_MOESM4_ESM.docx]

Description of Additional Supplementary Files

File Name: Supplementary Data 1
Description: Summary of retained introns. Summary of the values for intron retention and corresponding levels of significance after knockdown of Smu1, RED, or MFAP1.

File Name: Supplementary Data 2
Description: Alternative splicing events identified in ΔSmu1, ΔRED and ΔMFAP1 versus control cells. Column information about event coordinates, annotation and quantification is described in <https://github.com/ppapasaikas/SANJUAN>. Simple intron retention events are excluded.

File Name: Supplementary Data 3
Description: Protein Composition of Smu1/RED-depleted B complexes. Proteins of the indicated affinity-purified, human complexes were identified by LC-MS/MS after separation by SDS-PAGE. Proteins that were identified in both independent preparations (#1 and #2) are shown. Proteins not reproducibly detected or proteins generally considered to be common contaminants, such as ribosomal proteins, or those previously not detected in human spliceosomes are omitted. Shown are the total spectral counts for each protein. Proteins are grouped according to function or stage of recruitment. Note that kinetically-stalled B complexes typically contain small amounts of subsequently formed Bact/C complexes.
