# Supplementary material for: Smu1 and RED are required for activation of spliceosomal B complexes assembled on short introns
Source: Nat Commun. 2019 Aug 13;10:3639. doi: 10.1038/s41467-019-11293-8 (PMC6692369; doi:10.1038/s41467-019-11293-8)
Supplement: Supplementary file 2 — Peer Review [file 41467_2019_11293_MOESM2_ESM.pdf]

Reviewers' comments:

Reviewer #1 (Remarks to the Author):

NCOMMS-18-7828275
Smu1 and RED are required for the activation of spliceosomal B complexes assembled on short introns

Keiper, S., Panagiotis, P., Will, C.L., Valcárcel, J., Girard, C. and Luhrmann, R.

SMU1 and RED protein form a heterodimer that is associated with the spliceosomal B complex. They are absent in S. cerevisiae but highly conserved in all metazoans. Formation of the heterodimer is essential for the stability of both proteins. Loss of their function in cells has been observed to lead to changes in some forms of alternative splicing, consistent with their initial isolation. They can be modeled into cryo-EM structures of mammalian B complexes. They were identified as alternative splicing factors but are unusual in that they are also core components of the spliceosome. They do not appear to be essential under most conditions and this has led to a huge mystery in the splicing field; what is the function of these conserved proteins in splicing?

Initial experiments for this manuscript start with high-throughput sequencing analysis of changes in alternative splicing upon siRNA knockdown of SMU1 and RED, vs. another B-specific protein MFAP1. Here it was noted that these knockdowns lead to changes in different types of splicing events, including retained introns. Upon further examination, RED and SMU1 knockdowns showed a strong overlapping bias towards retention of short introns (<200nt), MFAP1 knockdown did not show this bias.

This led to a creative series of in vitro splicing, spliceosome assembly and depletion/reconstitution experiments on a series of intron deletion constructs. The dramatic result is that when intron size is reduced to 80 bases, SMU1 and RED are essential for splicing and B complex formation. This is the first demonstration of an essential role for these factors. The addition of both proteins are required for splicing and spliceosome assembly rescue. Using substrates containing a branch point but lacking a 3' exon (only capable of the first step of splicing) they demonstrate that the 5'ss to branchpoint distance is the determinant of the requirement for SMU1/RED dependent activity. There is a nice series of trans splicing experiments, in which in vitro splicing occurs between two separate RNAs; one containing the 5' exon and first part of the intron and the other containing the branchpoint and 3' exon. In this assay, the SMU1 and RED dependence of the splicing is no longer observed, even though the total amount of intron removed is short. This suggests a physical length constraint between 5'ss and branchpoint that is different in the absence of SMU1/RED.

This leads to a compelling model for the role of RED and SMU1 in the splicing of short introns. This is based on spliceosomal B complex cryoEM structures in figure 3C and drawn out in detain in figure 8. The model suggests that RED and SMU1 are required to lock the B spliceosome in a structure that is capable of splicing out short introns. In the absence of these proteins, bridging interactions between BRR2, SF3B3 and U2snRNP are not there and the spliceosome is too flexible to efficiently accommodate short distances between the branchpoint interaction with U2 and the 5' splice site interaction with U6. This flexibility leads to a drop in spliceosome efficiency, which is much more dramatic for shorter introns than longer introns.

I read this paper on a Friday, and as is my way, I then thought about it for a couple of days before writing this review. In a rare event in my career, I could not find any problems with the paper, only one minor suggestion. I truly think that this paper is groundbreaking for the field as it helps to explain a longstanding mystery about the role of this specific pair of B-specific proteins in splicing and alternative splicing. In that regard, it is a milestone paper. The intertwined use of bioinformatics, biochemistry approaches, biochemical depletion/reconstitution studies on B complex formation and in

vitro splicing, the studies with trans splicing substrates and then to tie this in with a model based on cryoEM models of B spliceosomes from senior author's laboratory, are a tour-de-force of careful modern science with a bedrock in classic splicing biochemistry. This huge toolkit allowed for the development of multiple testable hypotheses and an important breakthrough in the splicing field.

Minor comment:
My only minor comment is that in figures 2A and 5A, the convention used to show distances between splice sites and branchpoints look too much like the way we indicate alternative splicing (as in figure S1C). Perhaps use of a bar to show distances (like in mechanical drawings) would be better than the convention used in these drawings that look like alternative splicing diagrams.

Reviewer #2 (Remarks to the Author):

This study investigates the function of the human splicing factors Smu1 and RED. Although originally described as alternative splicing factors, these factors have been since been shown to be components of the spliceosome that associate transiently at the B complex stage, just prior to the activation of the spliceosome. Here, the authors reveal by a genome wide assay that Smu1 and RED play particularly important roles in splicing substrates with short introns. In vitro, the authors show that these factors are important for efficient splicing in general and essential for the splicing of short introns, and in an elegant experiment, the authors rescue the defect in splicing a short intron by separating the 5’ splice site and branch site. The authors also show evidence both in vivo and in vitro that these factors function at the stage of spliceosome activation. Although detailed mechanisms remain outstanding, the authors convincingly demonstrate the roles for Smu1 and RED in activating the spliceosome and in splicing short introns, through exceptionally well designed and executed experiments. This paper promises to be both well-read and well-regarded by those in the splicing field as well as those interested in the impact of genomic features on gene expression. The manuscript is appropriate for publication, though we recommend the authors consider the following points.

Specific comments:

1. Based on the comparative analysis of the MINX80 and PM5-116 substrates, which have similar 5’ splice site-BS distances but different BS-3’ss distances, the authors conclude that the 5’ splice site-BS distance, rather than the full intron length, matters; this may be true, but there are more variables changing here than intron length; sequence is also changing, so the authors need to qualify their conclusion accordingly, especially given this limited comparison. A more rigorous test would be to show that increasing the BS-3’ss distance in MINX80 by 10 or more nucleotides does not rescue splicing. On the other hand, if the 5’ splice site-BS distance, rather than the full intron length, is most key, then this dependency may be revealed in the genome-wide analysis. For example, it would be informative to examine whether the retained longer introns in Smu1/RED KD are more likely to have a short 5'SS-BS distance and whether the unaffected short introns in the KD experiment are more likely to have a long 5'SS-BS distance.

2. Page 6: “Analysis of spliceosome assembly revealed that depletion led to a … concomitant reduction in Bact and C complexes”. I see the reduction in C complex, but I do not see a reduction in Bact complexes for any of the substrates tested in this panel. Although this statement appears to be true for other panels in the paper and there is certainly no accumulation of the Bact complex, the authors should revise the statement relating to this panel.

3. With PM5-116, is the fold-decrease in splicing for the shorter intron really greater?

4. Figure 7a,b: the authors show that depleting B complex factors important for spliceosome

activation, especially Smu1, results in the unusual localization of P-SF3B1 to the soluble nuclear fraction, in addition to the chromatin fraction. The authors should note this and comment on whether this may reflect slow post-transcriptional activation of the spliceosome.

5. Given the purported implication of 5′ splice site-BS distance, it would be helpful to replot 1d and 1e in the supplement using 5′ splice site-BS distances, rather than intron distance, based on BS annotations from Pineda and Bradley (G&D, 2018).

6. In Figure 8, do the authors suggest that the SF3B3-Brr2 bridge that forms in yeast forms in mammals in the absence of Smu1/RED? If this interaction isn't normally functional in mammals, why would this interaction be conserved?

7. It seems worth noting that in Smu1/RED depleted extract, the B complex assembled on the MINX80 substrate appears stable, whereas the B complex assembled on the PM5-116 substrate is turned over.

8. To complement figure 1a, it would be helpful if the authors provide numbers of events for each affected class as a supplementary table.

9. Figure 1e would be improved if it also included intron retention vs retained intron length distribution for MFAP1 KD, as a comparison to Smu1/RED KD.

10. Although the authors proved the presence of both Smu1 and RED is required for MINX80 splicing, the authors should be cautious not to conclude that the interaction between Smu1 and RED is necessary; this would require an analysis of mutations that disrupted the interaction.

Reviewer #3 (Remarks to the Author):

Ms# 190562
Smu1 and RED are required for the activation of spliceosomal B complexes assembled on short introns
Sandra Keiper, Panagiotis Papasaikas, Cindy L. Will, Juan Valcárcel, Cyrille Girard, and Reinhard Lührmann

Keiper et al. analysed the functions of human B complex specific proteins, Smu1 and RED, by RNAi coupled with RNA-Seq. It turned out that those two factors are required for both alternative splicing and general splicing. Moreover, they found that Smu1 and RED are required for spliceosome activation especially for the pre-mRNAs that has short distance between 5′ splice site and branch site.
By using powerful in vitro splicing assay system, the authors obtained solid results to support their conclusion, and the results they show in the manuscript are scientifically so interesting.
I have some comments for the manuscript as follows in order to improve it.

1. Although the authors clearly demonstrate the function of Smu1-RED complex, I think it is required to show this complex functions in vivo for efficient splicing of 'short' introns. After knockdown in HeLa cells, the authors should try rescue experiments by checking splicing of some of the representative genes.

2. In Figure 6b, the schematic representations of RNAs (pre-mRNA and mRNA) are not aligned with corresponding bands.

3. In Figure S4e, the bottom panel is also marked as Smu1. I wonder this is Smu1ΔWD40.

3. It would be nicer to have a Figure that has schematic pictures for yeast B and human B complexes to compare them, since Smu1-RED are human specific.

MAX-PLANCK-INSTITUT FÜR BIOPHYSIKALISCHE CHEMIE
KARL-FRIEDRICH-BONHOEFFER-INSTITUT

MPI für biophysikalische Chemie, 37070 Göttingen

Abt. Zelluläre Biochemie
Prof. Dr. Reinhard Lührmann
Am Fassberg 11, D-37077 Göttingen
Tel.: (++49) (0)551-201-1405/1407
Fax: (++49) (0)551-201-1197
email: reinhard.luehrmann@mpi-bpc.mpg.de

Nature Communications

Manuscript NCOMMS-18-7828275A

12. April 2019

We would like to thank the reviewers for their very positive and constructive comments regarding our manuscript. We have followed the reviewer suggestions and restructured our manuscript accordingly. In the revised version, we have addressed the reviewer's comments as follows:

Reviewer #1

My only minor comment is that in figures 2A and 5A, the convention used to show distances between splice sites and branchpoints look too much like the way we indicate alternative splicing (as in figure S1C). Perhaps use of a bar to show distances (like in mechanical drawings) would be better than the convention used in these drawings that look like alternative splicing diagrams.

We have modified these figures such that the distances between splice sites, and between these and branch points, do not look like the typical representation of alternative splicing.

Reviewer #2

1. Based on the comparative analysis of the MINX80 and PM5-116 substrates, which have similar 5' splice site-BS distances but different BS-3'ss distances, the authors conclude that the 5' splice site-BS distance, rather than the full intron length, matters; this may be true, but there are more variables changing here than intron length; sequence is also changing, so the authors need to qualify their conclusion accordingly, especially given this limited comparison. A more rigorous test would be to show that increasing the BS-3'ss distance in MINX80 by 10 or more nucleotides does not rescue splicing.

We have increased the BS-3'ss distance in MINX80 by 10 nucleotides and show that this does not rescue splicing. This new data is described on p. 8 in the Results section and shown in Supplemental Fig. 4.

On the other hand, if the 5' splice site-BS distance, rather than the full intron length, is most key, then this dependency may be revealed in the genome-wide analysis. For example, it would be informative to examine whether the retained longer introns in Smu1/RED KD are more likely to have a short 5'SS-BS distance and whether the unaffected short introns in the KD experiment are more likely to have a long 5'SS-BS distance.

We have examined the 5'SS to BS distances using the recently published BS mapping data from Pineda and Bradley (Genes & Development, 2018). Analysis of these distances in introns retained or not-retained upon Smu1 / RED knockdown clearly shows that retained introns possess shorter 5'SS - BS distances than unaffected introns. That is, while most retained introns have an average 5'SS-BS distance of ca 60 nucleotides, clustering between 50 and 70 nucleotides, unaffected introns display a much wider distribution of longer distances (Figure 1d). Importantly, this is not the case for introns affected (or not affected) by MFAP1 knockdown (Figure 1d). We have restricted the presentation of our analysis to relatively short introns because of an inherent constraint of long introns to the analysis of 5' SS - BS distances. That is, while for long introns no apparent difference in 5'SS-BS distances is observed between introns retained upon Smu1/RED knockdown and non-retained ones, it is important to keep in mind that the distance between the BS and 3'SS is below 50 nucleotides in the vast majority of mammalian introns and therefore the 5'SS and BS are located far apart in long introns. This acutely complicates the rigorous comparison of 5'SS-BS distances, as intron lengths display enormous variation. For this reason, we have chosen not to include the analysis of 5'SS-BS distances in long introns in the revised version of the manuscript. Nevertheless, the analysis of relatively short introns clearly supports our conclusion that the 5'SS-BS distance is a key determinant of the dependence on Smu1 and RED for efficient splicing of an intron. We now include these data in Figure 1 and have moved some of the data previously shown in Figure 1 into Supplemental Figure 1. As the 5'SS-BS distances are now mentioned in the global analyses, we have also reorganized the text describing the *in vitro* splicing studies and focus directly on the differences in 5'SS-BS distance rather than on intron length.

2. Page 9: "Analysis of spliceosome assembly revealed that depletion led to a concomitant reduction in Bact and C complexes". I see the reduction in C complex, but I do not see a reduction in Bact complexes for any of the substrates tested in this panel. Although this statement appears to be true for other panels in the paper and there is certainly no accumulation of the Bact complex, the authors should revise the statement relating to this panel.

We have revised this sentence and deleted "and concomitant reduction in B^{act} and C complexes".

3. With PM5-116, is the fold-decrease in splicing for the shorter intron really greater?

Quantitation shows a clear trend towards less splicing efficiency of PM5-116 than PM5-211 at the 30, 60, 90 and 120 min time points.

4. Figure 7a,b: the authors show that depleting B complex factors important for spliceosome activation, especially Smu1, results in the unusual localization of P-SF3B1 to the soluble nuclear fraction, in addition to the chromatin fraction. The authors should note this and comment on whether this may reflect slow post-transcriptional activation of the spliceosome.

We now mention the enhanced P-SF3B1 signal in the soluble nuclear fraction after Smu1 knockdown in the legend to Figure 7. We have carried out a number of experiments to determine whether this is due to slow post-transcriptional activation of the spliceosome, and did not find any evidence that this is the case. It seems more likely that the P-SF3B1 signal in the soluble nuclear fraction is due to less stable, structurally compromised activated spliceosomes that may dissociate during the fractionation procedure and leak SF3B to the soluble fraction. However, due to space limitations and to the fact that this difference does not affect any of our general conclusions, we do not address this point in any detail in the revised version of the manuscript.

5. Given the purported implication of 5' splice site-BS distance, it would be helpful to replot 1d and 1e in the supplement using 5' splice site-BS distances, rather than intron distance, based on BS annotations from Pineda and Bradley (G&D, 2018).

We include a replot of the previous 1d (now 1c) in Supplemental Figure 1. It is important to note that there is a bias for long introns in the 5'SS-BS distances reported in Pineda and Bradley. That is, the 5'SS-BS distances could be determined for 58% of all introns but only for 45% of introns <250 nts and 27% for introns <100 nts. Thus, although the distributions of 5'SS-BS distances mirror those shown for intron length, there is a difference in the absolute values, due to less coverage of shorter introns with the former.

6. In Figure 8, do the authors suggest that the SF3B3-Brr2 bridge that forms in yeast forms in mammals in the absence of Smu1/RED? If this interaction isn't normally functional in mammals, why would this interaction be conserved?

Yes, we suggest that a similar (but not identical) bridge could form, but only if Smu1 and RED are absent, which is not normally the case within a cell. Furthermore, a direct SF3B3-Brr2 bridge may form at a later stage of human the splicing process – for example after release of Smu1 and RED. Thus, theoretically there is no apparent reason why a direct SF3B3-Brr2 bridge would not be able to function in the human system, albeit less efficiently, if the 5'SS-BS distance is sufficiently long.

7. It seems worth noting that in Smu1/RED depleted extract, the B complex assembled on the MINX80 substrate appears stable, whereas the B complex assembled on the PM5-116 substrate is turned over.

We now mention on p. 9 that the B complexes formed on PM5-116 pre-mRNA in Smu1/RED depleted extract is apparently less stable at longer time points than those formed on MINX-80.

8. To complement figure 1a, it would be helpful if the authors provide numbers of events for each affected class as a supplementary table.

We now include such a Table in Supplemental Materials.

9. Figure 1e would be improved if it also included intron retention vs retained intron length distribution for MFAP1 KD, as a comparison to Smu1/RED KD.

We now include the data for the MFAP1 knockdown. The data formerly in panel 1e has been moved to Supplemental Figure 1, to make room for the new 5'SS-BS distance data.

10. Although the authors proved the presence of both Smu1 and RED is required for MINX80 splicing, the authors should be cautious not to conclude that the interaction between Smu1 and RED is necessary; this would require an analysis of mutations that disrupted the interaction.

We agree that using Smu1 and/or RED mutants that would no longer interact with one another would provide definitive proof of this. Nonetheless our data are consistent with the idea that the interaction between Smu1 and RED is necessary for their function. We have tried to be careful about our conclusions by using the formulation “is consistent with the idea that”. We have also now removed this point from the discussion.

Reviewer #3

1. Although the authors clearly demonstrate the function of Smu1-RED complex, I think it is required to show this complex functions in vivo for efficient splicing of ‘short’ introns. After knockdown in HeLa cells, the authors should try rescue experiments by checking splicing of some of the representative genes.

We respectfully disagree with the reviewer on this point because (i) knockdown of Smu1 and of RED led to very similar splicing changes, strongly arguing that preferential retention of introns with short 5'SS-BS distances *in vivo* is not due to off-target effects of the individual subsets of siRNAs utilized, and (ii) *in vitro* complementation assays demonstrate the biochemical requirement of Smu1 and RED for splicing of short introns in a system in which these factors are specifically depleted from nuclear extracts.

2. In Figure 6b, the schematic representations of RNAs (pre-mRNA and mRNA) are not aligned with corresponding bands.

We have corrected this.

3. In Figure S4e, the bottom panel is also marked as Smu1. I wonder this is Smu1ΔWD40.

We have corrected this.

4. It would be nicer to have a Figure that has schematic pictures for yeast B and human B complexes to compare them, since Smu1-RED are human specific.

We now include a schematic of the yeast and human B complexes in Supplementary Figure 6.

We thank the reviewers for their helpful suggestions/comments and hope that the revised manuscript is now acceptable for publication in Nature Communications.

Best Regards,

Reinhard Lührmann
Cyrille Girard

REVIEWERS' COMMENTS:

Reviewer #1 (Remarks to the Author):

In reading the response to the reviewers and looking at the changes in the manuscript, I am satisfied with the changes that were made. I think that this will become a very important paper for the field.

Reviewer #2 (Remarks to the Author):

The authors have satisfactorily addressed all issues raised - even those that were optionally to address. The manuscript have improved as a consequence.

Reviewer #3 (Remarks to the Author):

The authors addressed the points I raised with the first round of the review. I have no further comments and requests before acceptance of the manuscript.
